# Supplementary material for: Uncovering the neural correlates of the urge-to-blink: A study utilising subjective urge ratings and paradigm free mapping
Source: Imaging Neurosci (Camb). 2025 Jul 18;3:IMAG.a.84. doi: 10.1162/IMAG.a.84 (PMC12330854; doi:10.1162/IMAG.a.84)
Supplement: Supplementary Material [file IMAG.a.84_supp.pdf]

Supplementary A: Temporal Signal-to-Noise Ratio (tSNR)

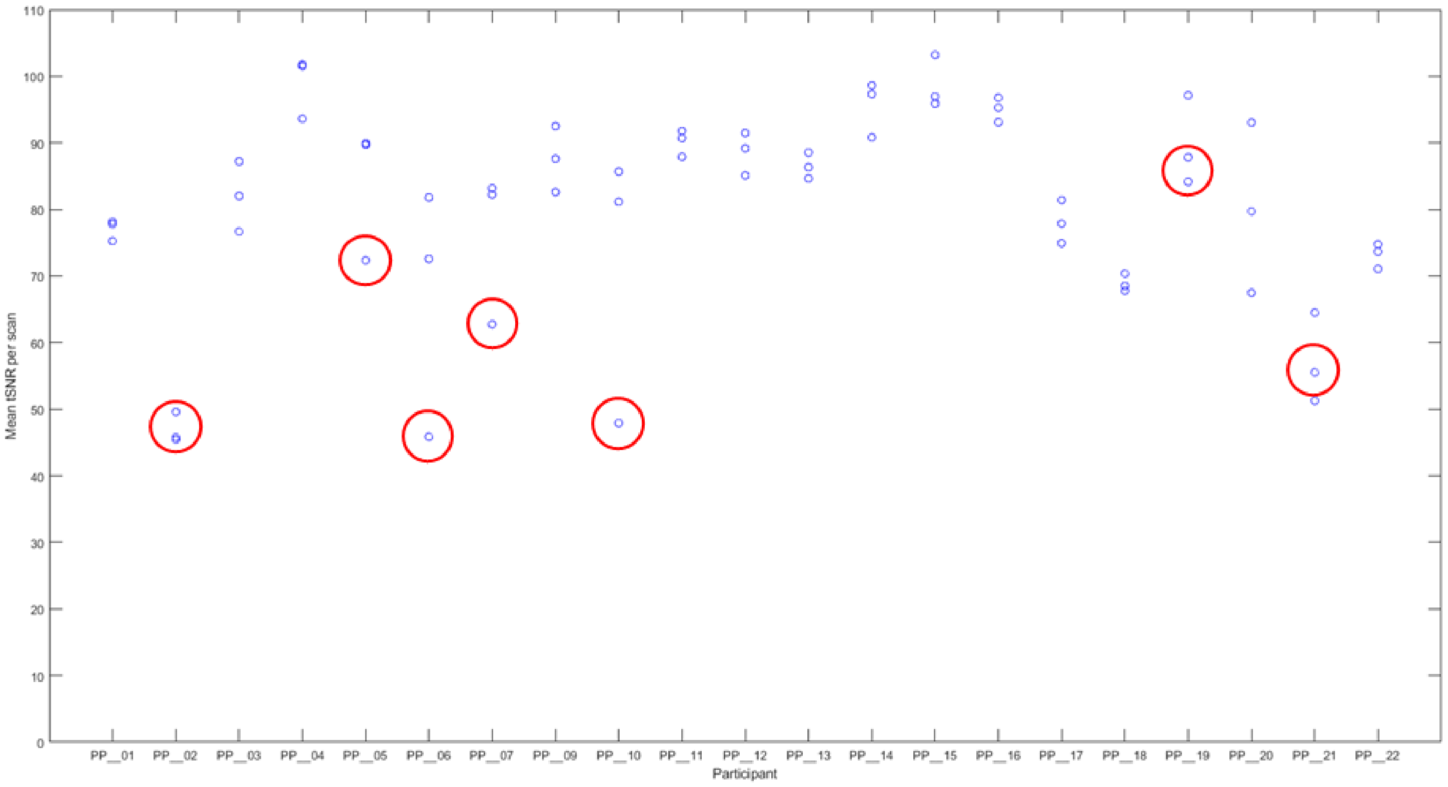

Figure SA.1. *A graph showing the mean tSNR for each fMRI run of the blink suppression paradigm, where scans encircled in red were excluded due to a maintained absolute mean displacement over 1.5mm. If found, scans with a tSNR below 30 would have been excluded.*

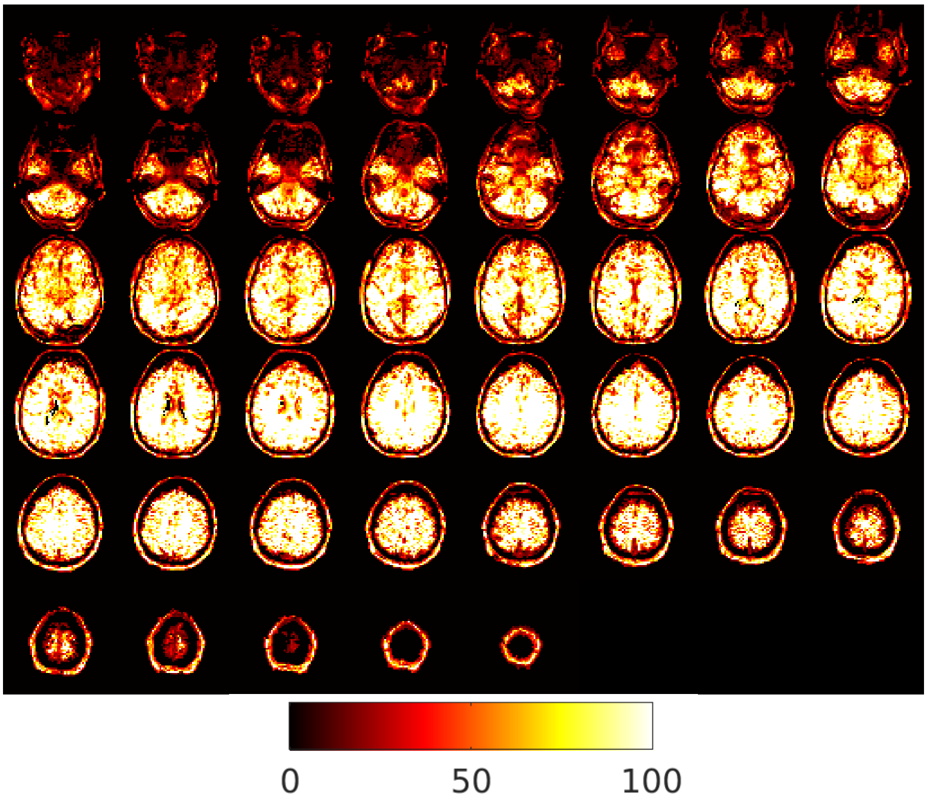

Figure SA.2*. An example (Sub01 run01) fMRI image with high tSNR.*

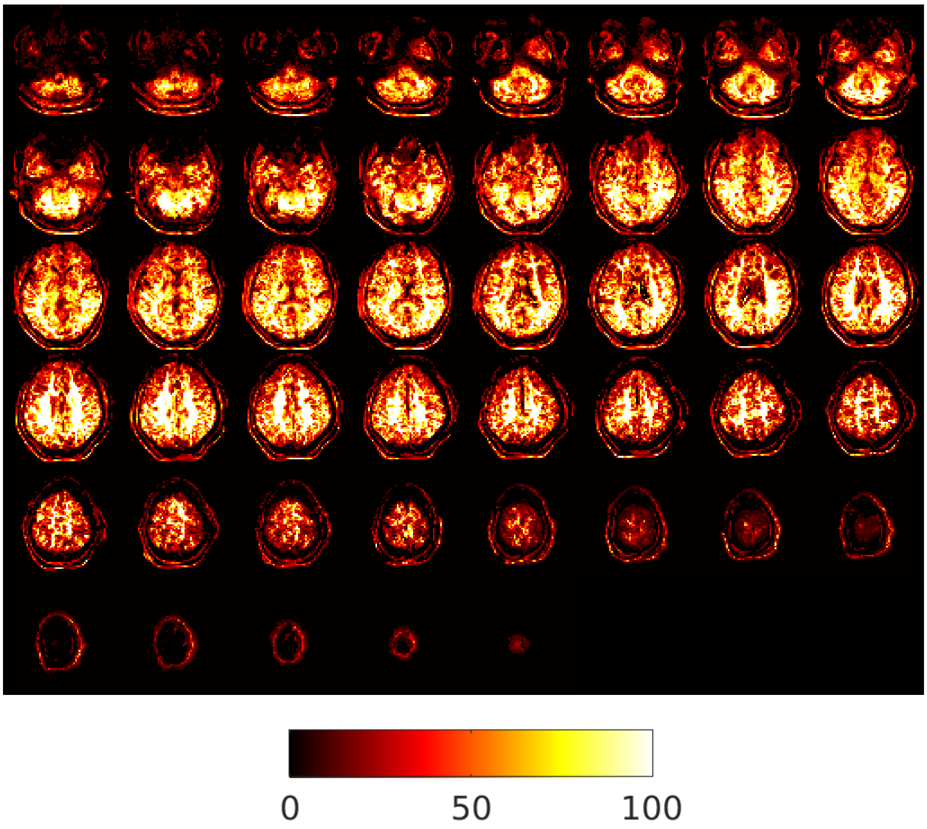

Figure SA.3. *An example (Sub02 run01) fMRI scan which was excluded due to a maintained absolute mean displacement over 1.5mm which caused a drop in tSNR.*

Supplementary B: Supplementary Methods

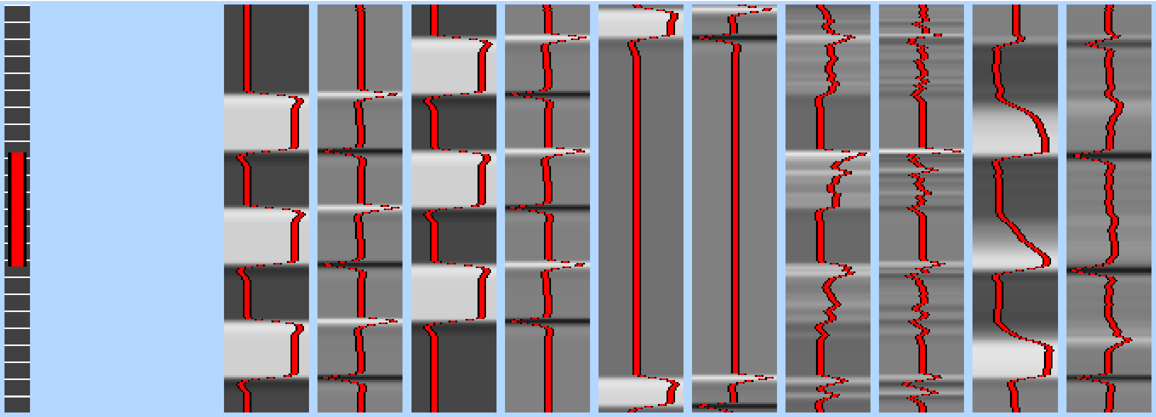

		Suppress	Suppress	Okay	Okay	Baseline	Baseline	Blinks	Blinks	Urge	Urge
C1	Suppress	1	0	0	0	0	0	0	0	0	0
C2	Okay	0	0	1	0	0	0	0	0	0	0
C3	Baseline	0	0	0	0	1	0	0	0	0	0
C4	Blinks	0	0	0	0	0	0	1	0	0	0
C5	Urge	0	0	0	0	0	0	0	0	1	0
C6	Suppress-Okay	1	0	-1	0	0	0	0	0	0	0
C7	Okay-Suppress	-1	0	1	0	0	0	0	0	0	0
C8	Baseline-Urge	0	0	0	0	1	0	0	0	-1	0
C9	Urge-Baseline	0	0	0	0	-1	0	0	0	1	0
C10	Urge-Blink	0	0	0	0	0	0	-1	0	1	0
C11	Blink-Urge	0	0	0	0	0	0	1	0	-1	0

Figure SB.1. *Figure showing a design matrix for a single run in a representative subject.*

Column 1 shows the timings of the 'Suppress' block, column 2 shows the temporal derivative.

Column 3 shows the timings of the 'Okay-to-blink' blocks, column 4 shows the temporal derivative.

Columns 5 shows the timings of the 'Random' baseline blocks, column 6 shows the temporal derivative.

Columns 7 shows the timings of blinks, column 8 shows the temporal derivative.

Columns 9 shows the parametric urge scores at each time point, column 10 shows the temporal derivative.

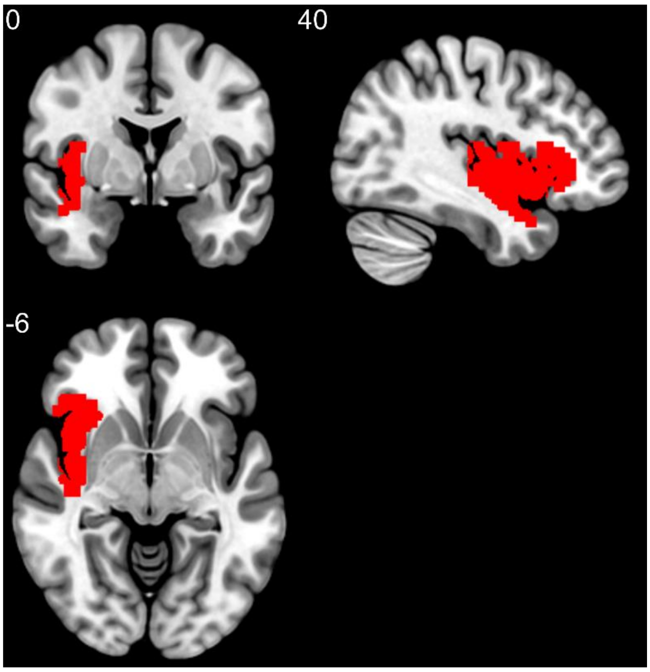

Figure SB.2. *Mask of the right insula used in the MESPFM analysis.*

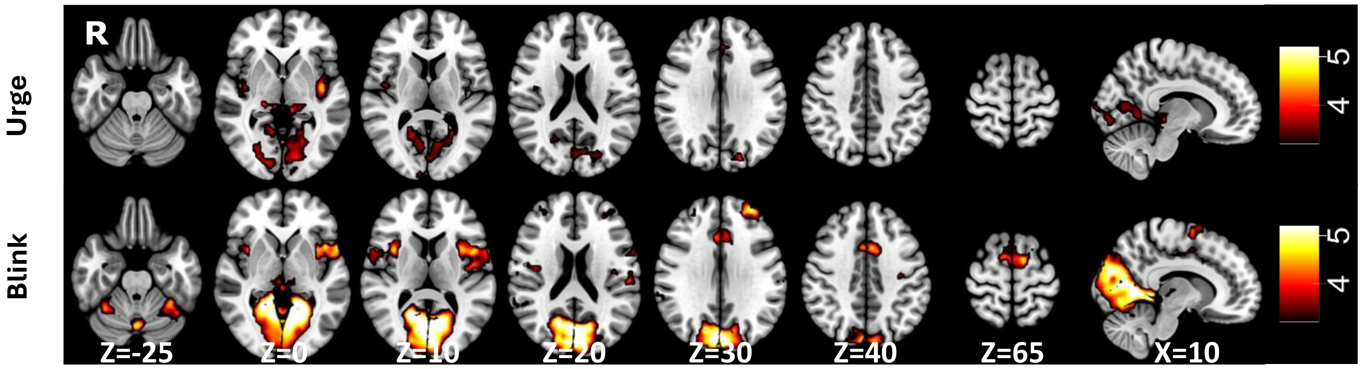

Figure SB.3. *BOLD response associated with the urge-to-blink and blinking.*

Statistical maps overlaid onto the MNI152 brain showing significant activations for the (top) 'Urge' and (bottom) 'Blink'. Statistical maps were thresholded at Z=3.2 (p<0.05).

Supplementary C: Local Maxima Cluster Index

Table SC.1. *Local maxima cluster index for 'Suppress' blocks.*

Cluster Size	Region	Z-score	MNI Coordinates		
			X	Y	Z
**Positive**					
2394	Left precentral gyrus	6.53	-36	-21	54
	Left postcentral gyrus	6.22	-57	-24	45
	Left precentral gyrus	6.18	-33	-12	66
	Left postcentral gyrus	6.15	-42	-33	60
	Left postcentral gyrus	6.12	-48	-27	45
	Left superior parietal lobule	5.74	-33	-48	57
1090	Right anterior supramarginal gyrus	6.3	57	-24	45
	Right postcentral gyrus	5.98	45	-33	51
	Right postcentral gyrus	5.8	36	-33	48
	Right superior lateral occipital cortex	5.71	18	-69	63
	Right postcentral gyrus	4.95	60	-18	33
	Right superior parietal lobule	4.41	39	-48	63
525	Right I-IV	6.41	6	-51	-15
	Right V	6.3	3	-57	-12
	Right V	6.28	15	-51	-18
	Vermis VIIIa	4.79	0	-69	-36
	Left I-IV	3.9	0	-45	-3
	Right crus I	3.31	42	-51	-27
442	Right inferior lateral occipital cortex	5.96	45	-66	6
	Right inferior lateral occipital cortex	4.89	51	-60	-6
	Right inferior lateral occipital cortex	4.69	45	-69	-6
	Right inferior lateral occipital cortex	3.62	33	-81	6
	Right inferior temporal gyrus	3.57	42	-48	-6
236	Left inferior lateral occipital cortex	6.2	-48	-75	6
	Left inferior lateral occipital cortex	5.02	-48	-63	6
217	Right precentral gyrus	5.49	57	12	27
	Right insular cortex	5.16	39	0	9
103	Left precentral gyrus	5.21	-54	3	36
	Left inferior frontal gyrus, pars opercularis	3.54	-54	9	18
	Left precentral gyrus	3.5	-60	12	21
61	Left thalamus	5.35	-15	-21	9
57	Left central opercular cortex	5.33	-39	-3	15
52	Left VI	4.26	-36	-36	-33
	Left VI	4.2	-33	-51	-27
	Left VI	3.9	-33	-39	-39
	Left VI	3.87	-24	-57	-21

Negative					
5690	Left cuneal cortex	7.52	-6	-87	30
	Right lingual gyrus	6.61	9	-60	3
	Right precuneous cortex	6.44	3	-54	15
	Left lingual gyrus	6.36	-18	-42	-6
	Left lingual gyrus	6.28	-12	-60	3
	Right cuneal cortex	6.21	3	-75	27
956	Right anterior superior temporal gyrus	5.7	57	-3	-12
	Right anterior middle temporal gyrus	5.29	63	-6	-9
	Right planum polare	5.21	60	0	3
	Right Heschl's gyrus	5.08	45	-12	0
	Right posterior superior temporal gyrus	4.91	66	-24	0
	Right anterior superior temporal gyrus	4.88	63	-6	0
544	Right frontal pole	4.89	21	60	0
	Left frontal pole	4.74	-6	63	3
	Left frontal pole	4.5	-6	57	3
	Right frontal pole	4.37	6	60	-3
	Right paracingulate gyrus	4.31	6	42	24
	Right frontal pole	4.29	12	66	0
202	Right angular gyrus	5.62	57	-57	27
	Right angular gyrus	4.49	51	-54	36
146	Left crus I	5.35	-21	-78	-33
	Left crus II	4.78	-9	-81	-33
	Left crus I	3.69	-42	-75	-36
109	Right superior frontal gyrus	4.32	21	33	39
	Right frontal pole	4.31	24	36	48
	Right middle frontal gyrus	4.04	27	30	48
	Right superior frontal gyrus	3.96	24	30	54
	Right middle frontal gyrus	3.89	27	27	42
	Right superior frontal gyrus	3.87	27	24	57
74	Left middle frontal gyrus	4.12	-27	15	48
	Left middle frontal gyrus	4.11	-27	21	51
	Left superior frontal gyrus	3.68	-18	27	39
	Left superior frontal gyrus	3.58	-24	18	39
	Left middle frontal gyrus	3.48	-27	27	45
55	Left IX	5.04	6	-45	-39
	Left IX	3.91	-6	-54	-39

Table SC.2*. Local maxima cluster index for 'Okay to blink' blocks.*

Cluster Size	Region	Z-score	MNI Coordinates		
			X	Y	Z
**Positive**					
1259	Left postcentral gyrus	5.73	-57	-27	48
	Left postcentral gyrus	5.46	-39	-36	54
	Left postcentral gyrus	5.39	-45	-27	45
	Left precentral gyrus	5.3	-42	-21	60
	Left postcentral gyrus	5.22	-36	-36	45
	Left superior parietal lobule	5.14	-33	-45	54
797	Right precentral gyrus	5.92	54	-21	42
	Right postcentral gyrus	5.56	42	-30	48
	Right superior lateral occipital cortex	4.8	18	-63	63
	Right superior parietal lobule	4.71	39	-48	63
460	Right inferior lateral occipital cortex	5.48	45	-69	6
	Right inferior lateral occipital cortex	4.94	54	-60	-6
	Right inferior lateral occipital cortex	4.45	45	-69	-6
	Right superior lateral occipital cortex	4.12	30	-84	9
	Right inferior temporal gyrus	4	45	-48	-6
	Right cerebral white matter (superior longitudinal fasciculus)	3.85	42	-51	3
212	Right precentral gyrus	5.07	33	-6	57
	Right precentral gyrus	3.63	15	-12	72
	Right supplementary motor area	3.24	12	-9	54
165	Left inferior lateral occipital cortex	6.23	-45	-72	6
	Left cerebral white matter (inferior longitudinal fasciculus)	3.84	-36	-63	9
135	Right V	4.7	12	-51	-18
	Right V	4.46	24	-42	-30
96	Right precentral gyrus	5.09	57	12	27
**Negative**					
4237	Left cuneal cortex	6.57	-6	-87	27
	Left lingual gyrus	6.42	-12	-60	3
	Left cuneal cortex	6.42	-9	-87	18
	Left intracalcarine cortex	6.22	-12	-75	15
	Right lingual gyrus	6.21	12	-60	3
	Right precuneous cortex	6.14	18	-57	9
903	Right central opercular cortex	5.46	60	-3	6
	Right anterior superior temporal gyrus	5	57	-3	-9
	Right temporal pole	4.65	54	6	-15
	Right planum polare	4.57	42	0	-15
	Right middle temporal gyrus	4.51	66	-39	3
	Right planum polare	4.49	63	-18	3

311	Right angular gyrus	5.49	57	-54	24
	Right angular gyrus	4.91	57	-54	36
	Right posterior supramarginal gyrus	3.82	48	-39	21
	Right angular gyrus	3.74	39	-51	21
	Right angular gyrus	3.59	54	-54	51
283	Right frontal pole	5.12	27	54	36
	Right frontal pole	4.54	18	72	6
	Right frontal pole	4.25	21	63	0
	Right frontal pole	4.2	30	48	30
	Right frontal pole	4.04	30	57	18
	Right frontal pole	4.02	27	45	39
175	Left insular cortex	4.54	-42	-6	-9
	Left planum temporale	4.34	-60	-9	3
	Left central opercular cortex	4.04	-51	-6	12
	Left insular cortex	4.01	-33	12	-18
	Left planum polare	4.01	-54	0	-6
	Left temporal pole	3.49	-30	6	-21
172	Anterior cingulate cortex	4.44	6	12	39
	Paracingulate gyrus	3.99	6	33	30
	Anterior cingulate cortex	3.96	-6	39	18
	Anterior cingulate cortex	3.95	0	33	24
	Anterior cingulate cortex	3.85	3	39	21
	Anterior cingulate cortex	3.42	6	45	6
162	Left crus I	5.65	-18	-78	-33
	Left crus I	3.9	-15	-90	-24
	Left VI	3.78	-9	-72	-24
	Vermis VI	3.58	3	-72	-21
102	Left posterior supramarginal gyrus	4.9	-54	-48	18
	Left middle temporal gyrus	3.97	-57	-48	6
	Left middle temporal gyrus	3.7	-66	-54	6
	Left posterior supramarginal gyrus	3.52	-57	-45	33
	Left planum temporale	3.36	-57	-33	15
30	Right middle frontal gyrus	3.9	39	27	33

Table SC.3*. Local maxima cluster index for 'Random' active baseline blocks.*

Cluster Size	Region	Z-score	MNI Coordinates		
			X	Y	Z
**Positive**					
1986	Left postcentral gyrus	6.26	-57	-24	45
	Left precentral gyrus	6.18	-36	-21	54
	Left postcentral gyrus	6.13	-42	-33	60
	Left precentral gyrus	6.03	-33	-12	63
	Left postcentral gyrus	6.01	-48	-27	45
	Left superior parietal lobule	5.73	-33	-48	57
939	Right anterior supramarginal gyrus	5.96	57	-24	45
	Right postcentral gyrus	5.75	42	-33	48
	Right postcentral gyrus	5.68	33	-36	48
	Right superior lateral occipital cortex	5.16	18	-66	63
	Right postcentral gyrus	5.12	60	-15	33
	Right superior parietal lobule	4.38	39	-48	60
500	Right inferior lateral occipital cortex	5.89	45	-69	6
	Right inferior lateral occipital cortex	4.91	51	-60	-6
	Right inferior lateral occipital cortex	4.59	45	-69	-6
	Right cerebral white matter (inferior longitudinal fasciculus)	4.4	39	-60	-3
	Right superior lateral occipital cortex	4.36	30	-84	9
	Right inferior temporal cortex	3.85	42	-48	-6
364	Right V	6.09	15	-51	-18
	Right I-IV	5.87	6	-51	-15
	Right V	5.59	3	-57	-12
	Left I-IV	3.4	0	-45	-3
205	Left inferior lateral occipital cortex	6.45	-48	-75	6
	Left inferior lateral occipital cortex	4.63	-48	-63	6
123	Right precentral gyrus	5.49	57	12	27
102	Left precentral gyrus	5.23	-54	3	33
66	Vermis VIIIa	4.7	3	-66	-36
50	Left thalamus	4.98	-15	-24	9
39	Left inferior lateral occipital cortex	4.14	-27	-90	6
36	Left central opercular cortex	5.19	-39	-3	15
**Negative**					
4473	Left cuneal cortex	7.33	-6	-87	30
	Left lingual gyrus	6.87	-12	-60	3
	Left intracalcarine cortex	6.73	-6	-75	15
	Right cuneal cortex	6.71	3	-75	27
	Left lingual gyrus	6.63	-18	-42	-6
	Right lingual gyrus	6.58	12	-60	3

1007	Right central opercular cortex	5.95	60	0	6
	Right anterior superior temporal gyrus	5.54	57	0	-15
	Right temporal pole	4.99	45	9	-27
	Right Heschl's gyrus	4.95	45	-12	0
	Right planum polare	4.86	45	-3	-12
	Right temporal pole	4.85	45	15	-15
571	Right paracingulate gyrus	4.54	6	42	21
	Right frontal pole	4.44	21	60	0
	Right paracingulate gyrus	4.29	12	42	18
	Right frontal pole	4.27	24	54	12
	Right frontal pole	4.19	24	57	27
	Right frontal pole	4.13	24	54	-3
313	Right angular gyrus	5.72	57	-54	24
	Right angular gyrus	4.87	51	-54	36
	Right angular gyrus	3.88	54	-54	51
	Right angular gyrus	3.78	39	-51	21
	Right posterior supramarginal gyrus	3.49	48	-39	21
279	Left planum polare	4.69	-51	-3	-6
	Left insular cortex	4.59	-42	-3	-12
	Left planum polare	4.38	-57	-9	3
	Left frontal orbital cortex	4.25	-33	12	-21
	Left anterior superior temporal gyrus	4.24	-63	-12	0
	Left anterior superior temporal gyrus	4.1	-54	-12	-6
240	Left angular gyrus	5.2	-54	-51	18
	Left posterior superior temporal gyrus	4.13	-48	-33	3
	Left planum polare	3.98	-60	-33	15
	Left posterior supramarginal gyrus	3.97	-63	-48	21
	Left superior lateral occipital cortex	3.85	-57	-63	21
	Left posterior middle temporal gyrus	3.7	-45	-42	3
229	Right middle frontal gyrus	4.59	39	9	45
	Right superior frontal gyrus	4.43	21	33	39
	Right middle frontal gyrus	4.17	27	33	48
	Right superior frontal gyrus	3.96	24	24	51
	Right middle frontal gyrus	3.9	36	12	33
	Right superior frontal gyrus	3.88	27	24	57
152	Left crus I	5.38	-21	-81	-30
	Left crus II	4.96	-9	-78	-33
64	Left insular cortex	4.06	-36	18	-3
	Left insular cortex	3.97	-27	27	0
	Left frontal operculum cortex	3.64	-30	27	9
	Left putamen	3.55	-21	18	-3
	Left cerebral white matter (inferior fronto-occipital fasciculus)	3.49	-24	27	12

50	Left crus I	4.13	-42	-66	-42
	Left crus II	3.87	-36	-72	-42
	Left crus I	3.8	-42	-75	-36
	Left crus I	3.73	-54	-63	-30
49	Right IX	4.59	3	-45	-42
	Left IX	3.84	-9	-51	-33
31	Right thalamus	4.07	6	-9	12
	Right thalamus	3.52	3	-21	3
	Left thalamus	3.41	-3	-6	6

Table SC.4. *Local maxima cluster index for blinks.*

Cluster Size	Region	Z-score	MNI Coordinates		
			X	Y	Z
**Positive**					
4484	Left lingual gyrus	6.39	-21	-51	-3
	Left intracalcarine cortex	6.37	-12	-72	12
	Left intracalcarine cortex	6.29	-12	-63	6
	Right lingual gyrus	6.16	15	-45	-3
	Right lingual gyrus	6.1	21	-51	-6
	Right intracalcarine cortex	5.87	15	-66	15
637	Left superior frontal gyrus	4.98	-12	-3	66
	Anterior cingulate cortex	4.6	6	12	39
	Anterior cingulate cortex	4.56	3	18	36
	Anterior cingulate cortex	4.52	-6	12	39
	Left superior frontal gyrus	4.48	-15	6	72
	Left supplementary motor area	4.31	-6	0	54
588	Left precentral gyrus	5.96	-54	6	3
	Left central opercular cortex	5.38	-45	6	6
	Left central opercular cortex	4.92	-36	9	15
	Left insular cortex	4.74	-36	9	3
	Left postcentral gyrus	4.7	-57	-24	21
	Left central opercular cortex	4.62	-42	-9	9
263	Right insular cortex	4.94	36	9	9
	Right central opercular cortex	4.29	54	-6	15
	Right precentral gyrus	4.19	57	6	6
	Right central opercular cortex	4.18	48	6	6
	Right precentral gyrus	3.87	51	3	12
	Right central opercular cortex	3.86	42	-12	21
149	Left frontal pole	5.08	-30	51	30
	Left frontal pole	4.73	-42	48	27
	Left frontal pole	4.66	-36	42	24
	Left frontal pole	4.46	-30	45	21
67	Left precentral gyrus	4.7	-39	-9	51
	Left postcentral gyrus	4.34	-36	-18	39
	Left precentral gyrus	3.58	-51	-6	54
49	Right angular gyrus	4.02	54	-51	15
	Right posterior supramarginal gyrus	3.99	60	-39	27
	Right angular gyrus	3.65	48	-48	24
39	Right precentral gyrus	3.81	48	0	54
	Right precentral gyrus	3.7	42	-6	57
	Right precentral gyrus	3.7	45	0	48
36	Right frontal pole	4.2	36	51	21

	Right frontal pole	3.85	30	48	39
	Right frontal pole	3.62	27	45	30
	Right frontal pole	3.28	24	48	24
**Negative**					
85	Right angular gyrus	4.46	36	-54	39
	Right superior lateral occipital cortex	4.1	33	-57	66
	Right superior lateral occipital cortex	3.75	39	-60	60
	Right superior parietal lobule	3.52	39	-45	48
	Right superior parietal lobule	3.51	36	-39	48

Table SC.5. *Local maxima cluster index related to the subjective urge ratings.*

Cluster Size	Region	Z-score	MNI Coordinates		
			X	Y	Z
**Positive**					
1317	Right anterior thalamic radiation	4.91	3	-30	3
	Left thalamus	4.61	-6	-33	3
	Left intracalcarine cortex	4.49	-12	-72	15
	Left lingual gyrus	4.42	-12	-78	-3
	Right occipital pole	4.35	9	-99	9
	Right thalamus	4.32	18	-27	0
110	Left insular cortex	4.58	-42	-9	0
	Left insular cortex	4.52	-39	-15	6
69	Right planum polare	4.02	48	-9	-3
	Right insular cortex	3.99	39	-9	0
	Right planum polare	3.78	48	3	-3
	Right Heschl's gyrus	3.73	42	-18	3
	Right planum polare	3.49	45	-3	-9
50	Left insular cortex	3.9	-30	12	-12
	Left putamen	3.88	-21	6	-9
	Left putamen	3.36	-15	12	-9
41	Paracingulate gyrus	3.98	-3	33	33
	Anterior cingulate gyrus	3.87	0	24	33
37	Right central opercular cortex	3.79	48	-6	9
	Right insular cortex	3.66	33	-12	18
**Negative**					
49	Right superior lateral occipital cortex	4.29	18	-75	57
	Right superior lateral occipital cortex	3.89	27	-72	57
	Right precuneous cortex	3.47	9	-69	54
42	Right posterior supramarginal gyrus	4.18	39	-45	39
	Right posterior supramarginal gyrus	3.99	36	-39	36
33	Left superior lateral occipital cortex	3.96	-21	-63	51
	Left precuneous cortex	3.68	-6	-69	51

Table SC.6. *Local maxima cluster index when contrasting 'Suppress' > 'Okay to blink' blocks.*

Cluster Size	Region	Z-score	MNI Coordinates		
			X	Y	Z
721	Right superior frontal gyrus	4.81	27	12	66
	Anterior cingulate cortex	4.62	-6	21	30
	Right superior frontal gyrus	4.62	6	12	63
	Paracingulate gyrus	4.53	0	12	45
	Paracingulate gyrus	4.5	-9	15	42
	Right superior frontal gyrus	4.38	12	6	66
459	Vermis VIIIa	5.06	-3	-69	-36
	Vermis VIIIa	4.6	-3	-63	-33
	Left I-IV	4.53	0	-45	-15
	Left VI	4.52	-6	-72	-12
	Right V	4.33	6	-60	-6
	Vermis VI	4.27	0	-72	-12
362	Left crus I	4.85	-54	-51	-36
	Left VI	4.56	-36	-57	-27
	Left VI	4.49	-27	-57	-21
	Left VI	4.46	-18	-72	-18
	Left crus I	4.43	-48	-45	-36
	Left VI	4.43	-24	-66	-27
337	Right frontal operculum	4.92	36	21	9
	Right inferior frontal gyrus, pars opercularis	4.76	48	6	15
	Right frontal operculum cortex	4.68	42	15	9
	Right insular cortex	4.13	36	6	3
	Right inferior frontal gyrus, pars opercularis	3.9	57	12	9
	Rightinferior frontal gyrus, pars opercularis	3.88	54	15	21
281	Right posterior SMG	4.33	60	-39	33
	Right posterior SMG	4.1	48	-42	60
	Right posterior SMG	4.05	66	-39	27
	Right middle temporal gyrus	4.03	45	-54	12
	Right angular gyrus	3.93	48	-48	21
	Right anterior supramarginal gyrus	3.89	54	-30	39
250	Left superior lateral occipital	4.26	-18	-63	63
	Left superior parietal lobule	4.23	-27	-54	60
	Left superior lateral occipital cortex	4.18	-12	-63	63
	Left superior lateral occipital	4.12	-15	-60	54
	Left superior lateral occipital	4.09	-18	-63	45
	Left superior lateral occipital	4.09	-15	-75	48
236	Left frontal operculum	4.7	-36	12	12
	Left frontal operculum	4.69	-33	18	12

	Left precentral gyrus	4.12	-54	6	9
	Left inferior frontal gyrus, pars opercularis	4.02	-57	12	0
	Left central opercular cortex	3.91	-48	-3	6
193	Right frontal pole	5.72	36	57	21
	Right frontal pole	4.6	27	57	30
	Right frontal pole	4.14	33	48	39
188	Right superior lateral occipital	4.29	15	-60	54
	Right superior lateral occipital	4.15	12	-72	48
	Right superior lateral occipital	3.96	9	-60	72
	Right superior lateral occipital	3.78	15	-72	63
	Right precuneous cortex	3.64	18	-66	42
	Right superior parietal lobule	3.49	24	-54	51
120	Right cerebellum	4.81	21	-45	-42
	Right VI	4.25	39	-45	-33
	Right VI	4.15	30	-51	-30
	Right VI	3.77	30	-39	-33
	Right crus II	3.65	33	-48	-42
109	Left superior frontal gyrus	4.45	-24	3	57
	Left superior frontal gyrus	3.93	-12	-6	72
	Left superior frontal gyrus	3.91	-18	6	69
	Left superior frontal gyrus	3.59	-27	-6	72
91	Left frontal pole	4.48	-36	45	18
	Left frontal pole	3.99	-33	51	24
	Left frontal pole	3.95	-36	51	30
	Left frontal pole	3.91	-33	57	21
60	Left inferior lateral occipital	4.56	-45	-78	12
	Left inferior lateral occipital	3.57	-54	-66	12
	Left middle temporal gyrus	3.55	-45	-60	6
59	Left precentral gyrus	4.3	-12	-21	42
	Left precentral gyrus	4.25	-15	-33	45
	Left postcentral gyrus	3.86	-18	-42	54
42	Posterior cingulate cortex	4.24	9	-30	45
	Posterior cingulate cortex	4.21	6	-21	45
	Right precuneous cortex	3.67	15	-36	45
	Right postcentral gyrus	3.56	21	-42	51

Table SC.7. *Local maxima cluster index when contrasting 'Okay to blink' > 'Suppress' blocks.*

Cluster Size	Region	Z-score	MNI Coordinates X	Y	Z
261	Posterior cingulate cortex	5	3	-48	21
	Posterior cingulate cortex	4.25	-9	-51	21
	Posterior cingulate cortex	4.04	0	-42	36
	Posterior cingulate cortex	4.02	-6	-45	12
	Left precuneous cortex	3.84	-3	-60	36
207	Left middle frontal gyrus	4.6	-27	18	48
	Left middle frontal gyrus	4.58	-33	18	54
	Left superior frontal gyrus	4.43	-15	24	48
	Left superior frontal gyrus	4.39	-21	27	48
	Left frontal lobe	3.86	-15	45	54
	Paracingulate gyrus	3.84	-3	39	36
203	Left superior lateral occipital	4.63	-42	-72	42
	Left superior lateral occipital	4.52	-39	-63	39
	Left superior lateral occipital	4.39	-45	-72	33
	Left superior lateral occipital	4.33	-36	-72	48
	Left angular gyrus	4.12	-42	-60	33
52	Frontal pole	4.55	0	63	-6
	Right frontal pole	4.48	6	63	-6
52	Left hippocampus	3.77	-36	-18	-12
	Left parahippocampal gyrus	3.58	-27	-33	-12
	Left amygdala	3.55	-27	-9	-18
51	Left frontal orbital cortex	4.61	-48	36	-9
	Left frontal pole	4.03	-42	45	-6
44	Right frontal pole	4.04	18	36	48
	Right superior frontal gyrus	3.79	18	36	57
42	Right crus I	4.04	27	-81	-24
	Right crus II	3.93	24	-84	-36
	Right crus I	3.84	30	-84	-30
	Right crus I	3.67	39	-81	-27
	Right crus II	3.54	15	-84	-36

Table SC.8. *Local maxima cluster index when contrasting 'Random' > Urge blocks.*

Cluster Size	Region	Z-score	MNI Coordinates		
			X	Y	Z
1660	Left precentral gyrus	6.53	-36	-21	54
	Left postcentral gyrus	6.37	-57	-24	45
	Left postcentral gyrus	6.19	-42	-33	60
	Left postcentral gyrus	6.19	-48	-27	45
	Left precentral gyrus	6.07	-33	-12	63
	Left superior parietal lobule	5.76	-33	-48	57
953	Right anterior SMG	6.26	57	-24	45
	Right postcentral gyrus	5.78	45	-33	51
	Right postcentral gyrus	5.72	36	-33	48
	Right superior lateral occipital	5.47	18	-69	63
	Right postcentral gyrus	4.84	60	-15	33
	Right superior parietal lobule	4.32	39	-48	60
437	Right inferior lateral occipital	6.02	45	-69	6
	Right inferior temporal gyrus	4.96	51	-57	-6
	Right inferior temporal gyrus	4.13	42	-48	-6
	Right superior lateral occipital	3.78	30	-84	9
386	Right V	6.48	15	-51	-18
	Right I-IV	6.3	6	-51	-15
	Right VI	3.83	15	-63	-21
	Left I-IV	3.59	0	-45	-3
386	Right precentral gyrus	5.05	27	-9	51
	Right precentral gyrus	4.82	27	-9	57
	Right precentral gyrus	4.75	27	-12	63
	Right precentral gyrus	4.67	33	-12	60
	Right superior frontal gyrus	4.43	24	3	60
	Right precentral gyrus	3.6	15	-9	63
205	Left inferior lateral occipital	6.47	-45	-72	6
	Left inferior lateral occipital	4.87	-48	-63	6
120	Right precentral gyrus	5.56	57	12	27
85	Left precentral gyrus	5.19	-54	3	36
72	Vermis VIIIa	4.72	3	-66	-36
47	Left thalamus	4.93	-18	-21	9
31	Left central opercular cortex	5.07	-39	-3	15

Table SC.9. *Local maxima cluster index when contrasting Urge > 'Random' blocks.*

Cluster Size	Region	Z-score	MNI Coordinates		
			X	Y	Z
5595	Left cuneal cortex	8.05	-6	-87	30
	Left lingual gyrus	6.87	-18	-42	-6
	Left lingual gyrus	6.8	-12	-60	3
	Left intracalcarine cortex	6.79	-6	-75	15
	Right cuneal cortex	6.73	3	-75	27
	Right lingual gyrus	6.68	9	-60	3
1176	Right central opercular cortex	5.99	60	0	6
	Right anterior superior temporal gyrus	5.69	57	0	-15
	Right Heschl's gyrus	5.21	45	-12	0
	Right temporal pole	5.05	45	9	-27
	Right temporal pole	4.93	45	15	-15
	Right temporal pole	4.91	36	6	-18
665	Paracingulate cortex	4.76	6	42	21
	Paracingulate cortex	4.57	0	42	27
	Right frontal pole	4.49	21	60	0
	Anterior cingulate cortex	4.42	-3	33	24
	Paracingulate cortex	4.38	12	42	18
	Left frontal pole	4.33	-6	57	3
291	Right angular gyrus	5.52	57	-54	24
	Right angular gyrus	4.74	51	-54	36
	Right angular gyrus	3.76	39	-51	21
	Right angular gyrus	3.67	45	-45	21
	Right angular gyrus	3.66	54	-54	51
	Right posterior supramarginal gyrus	3.66	48	-39	21
132	Left Crus I	5.25	-21	-78	-33
	Left Crus II	4.65	-9	-78	-33
94	Right superior frontal gyrus	4.13	21	33	39
	Right middle frontal gyrus	4.06	27	33	48
	Right superior frontal gyrus	3.7	27	24	57
	Right superior frontal gyrus	3.62	24	24	51
61	Right IX	5.14	3	-45	-42
	Left IX	4.22	-6	-51	-33
47	Right middle frontal gyrus	4.87	39	9	45
	Right middle frontal gyrus	3.95	36	12	33
	Right middle frontal gyrus	3.36	42	24	33
	Right middle frontal gyrus	3.25	45	24	42

Table SC.10. *Local maxima cluster index when contrasting Blinks > Urge blocks.*

Cluster Size	Region	Z-score	MNI Coordinates X	Y	Z
4149	Left lingual gyrus	6.18	-21	-51	-3
	Left intracalcerine cortex	6.05	-12	-63	6
	Left intracalcerine cortex	6.05	-12	-72	12
	Right lingual gyrus	5.98	15	-45	-3
	Left lingual gyrus	5.77	-9	-57	0
	Right precuneous cortex	5.74	18	-60	12
538	Left superior frontal gyrus	4.97	-12	-3	66
	Anterior cingulate cortex	4.52	-6	12	39
	Left superior frontal gyrus	4.45	-18	6	72
	Anterior cingulate cortex	4.42	3	12	39
	Anterior cingulate cortex	4.36	3	18	36
	Right superior frontal gyrus	4.29	12	0	63
502	Left central opercular cortex	5.5	-45	6	6
	Left precentral gyrus	5.45	-57	6	3
	Left frontal opercular cortex	5.15	-45	12	0
	Left postcentral gyrus	5.12	-57	-24	21
	Left frontal opercular cortex	4.92	-36	12	15
	Left insular cortex	4.6	-36	9	3
201	Right insular cortex	4.73	36	9	9
	Right central opercular cortex	4.24	54	-6	15
	Right precentral gyrus	4.03	57	6	6
	Right central opercular cortex	3.94	48	6	6
	Right precentral gyrus	3.84	51	3	12
	Right central opercular cortex	3.66	42	-12	21
138	Left frontal pole	5.07	-30	51	30
	Left frontal pole	4.73	-30	45	21
	Left frontal pole	4.59	-42	48	27
	Left frontal pole	4.22	-24	60	33
54	Left postcentral gyrus	4.71	-36	-18	39
	Left precentral gyrus	4.68	-42	-9	51
37	Right frontal pole	4.43	36	51	21
	Right frontal pole	3.89	30	48	36
	Right frontal pole	3.68	27	45	30

Supplementary D: Activation Time Series

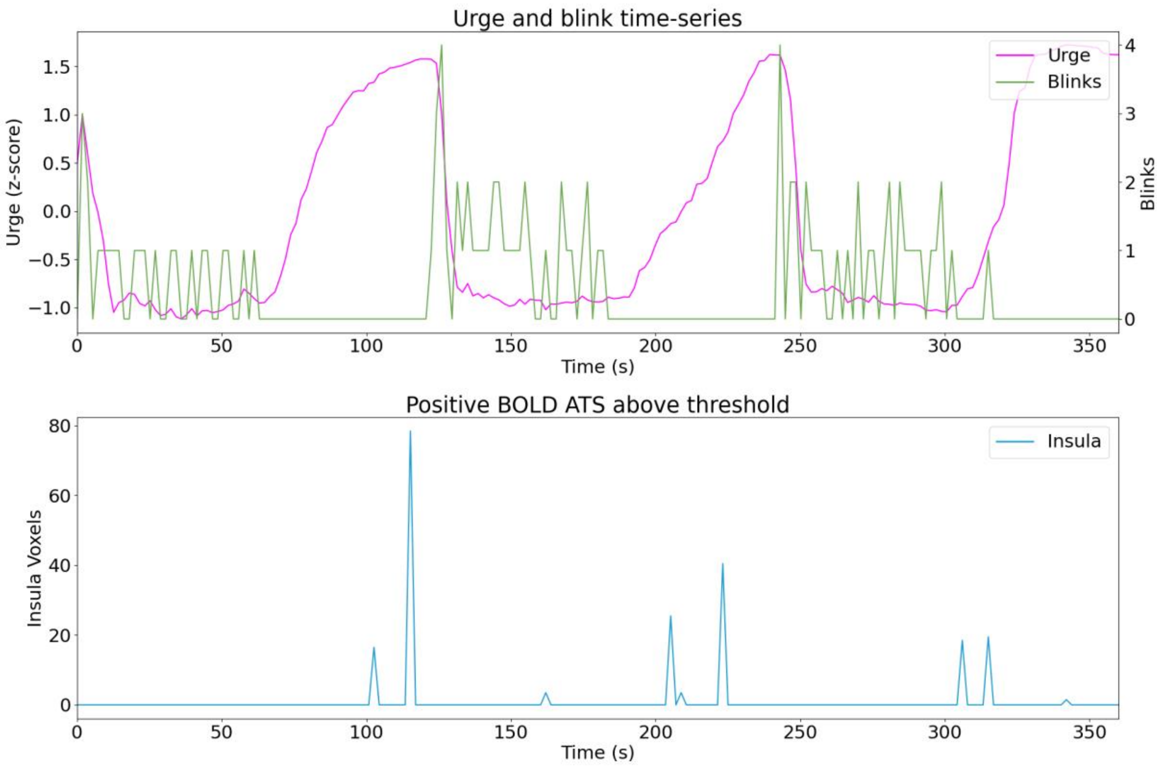

Figure SD.1. *Sub01 run01*

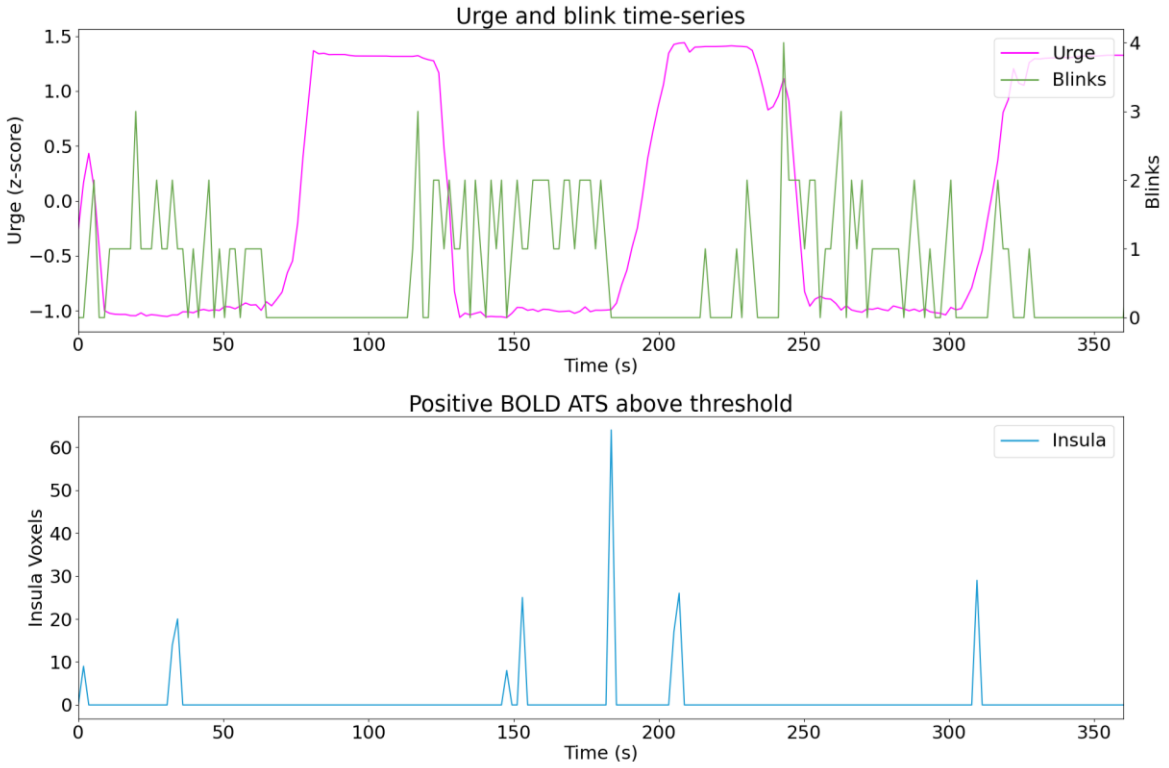

Figure SD.2. *Sub01 run02*

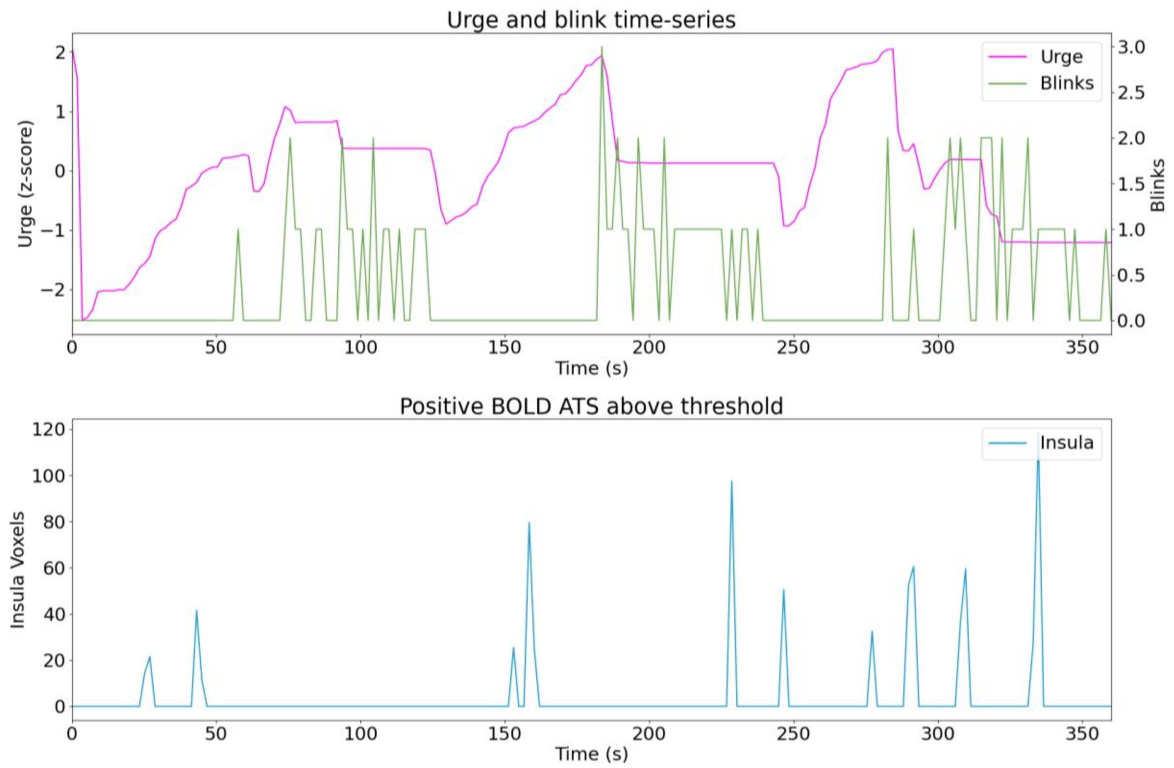

Figure SD.3. *Sub03 run01*

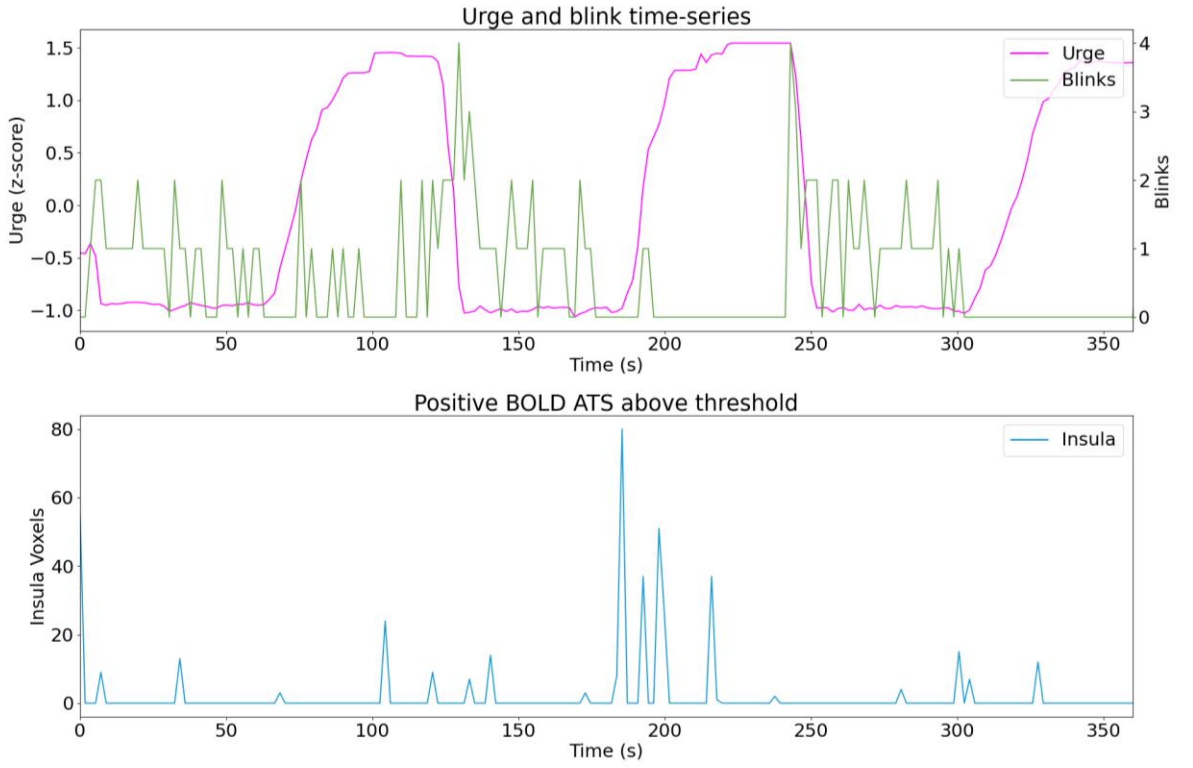

Figure SD.4. *Sub01 run03*

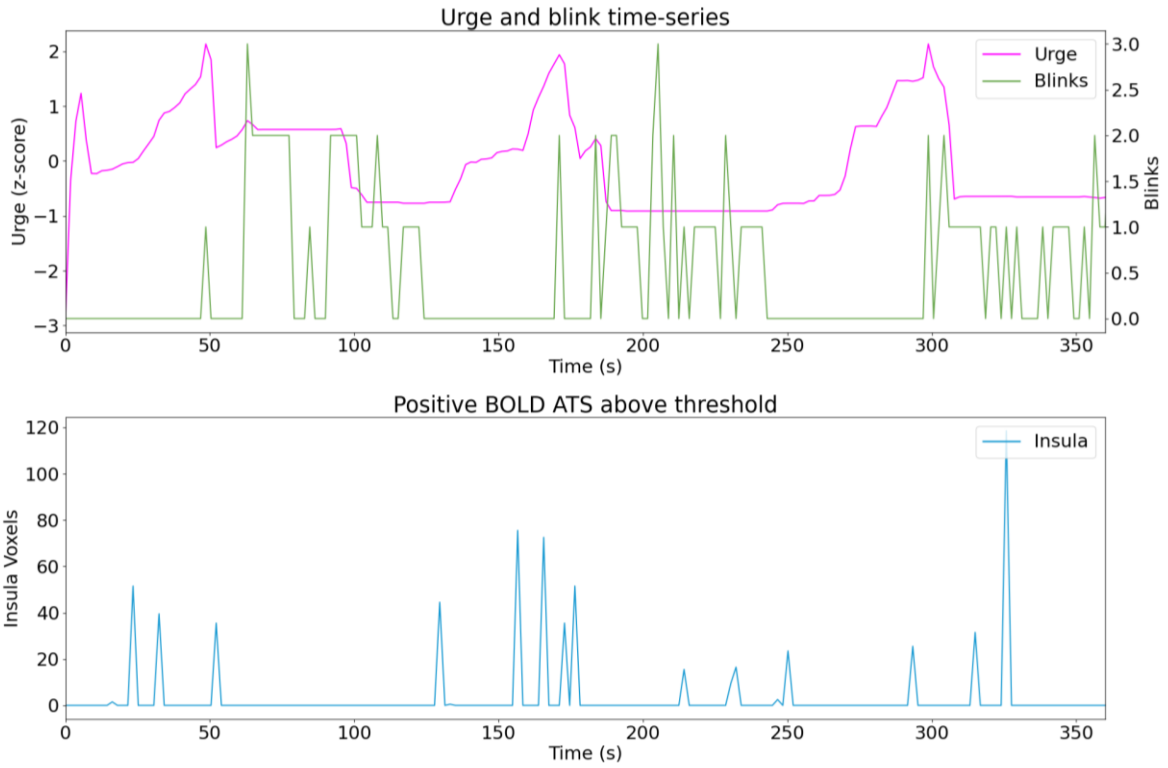

Figure SD.5. *Sub03 run03*

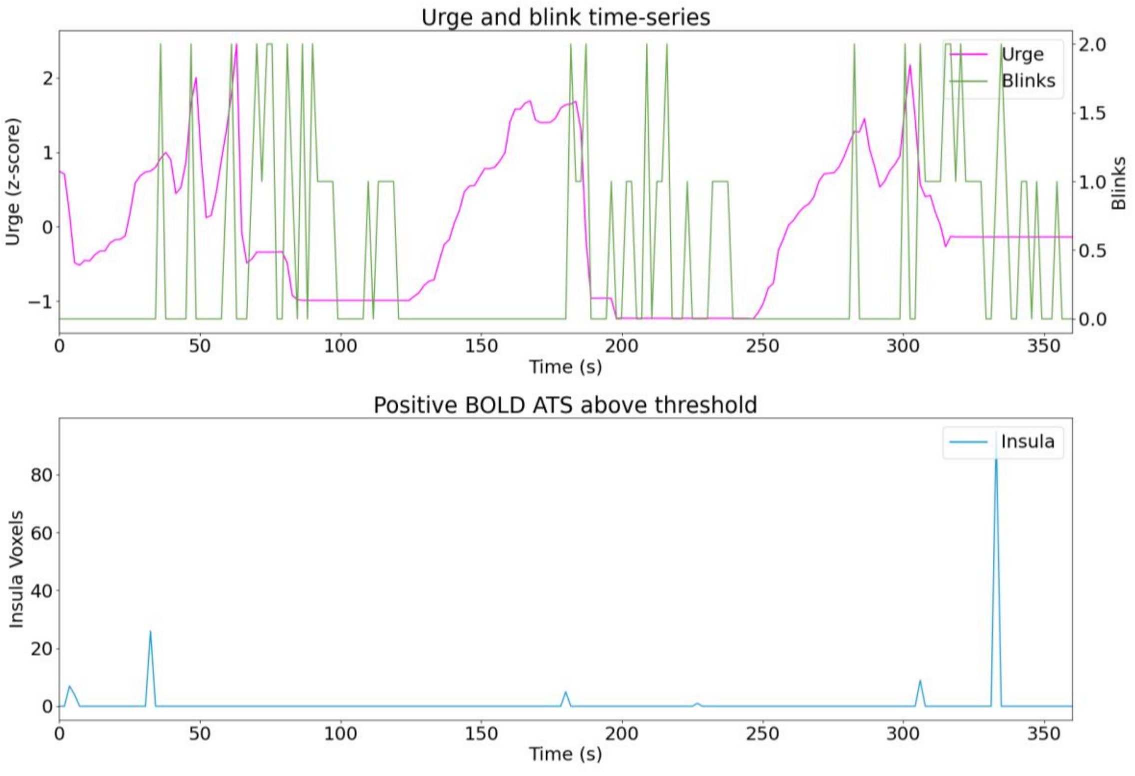

Figure SD.6. *Sub03 run02*

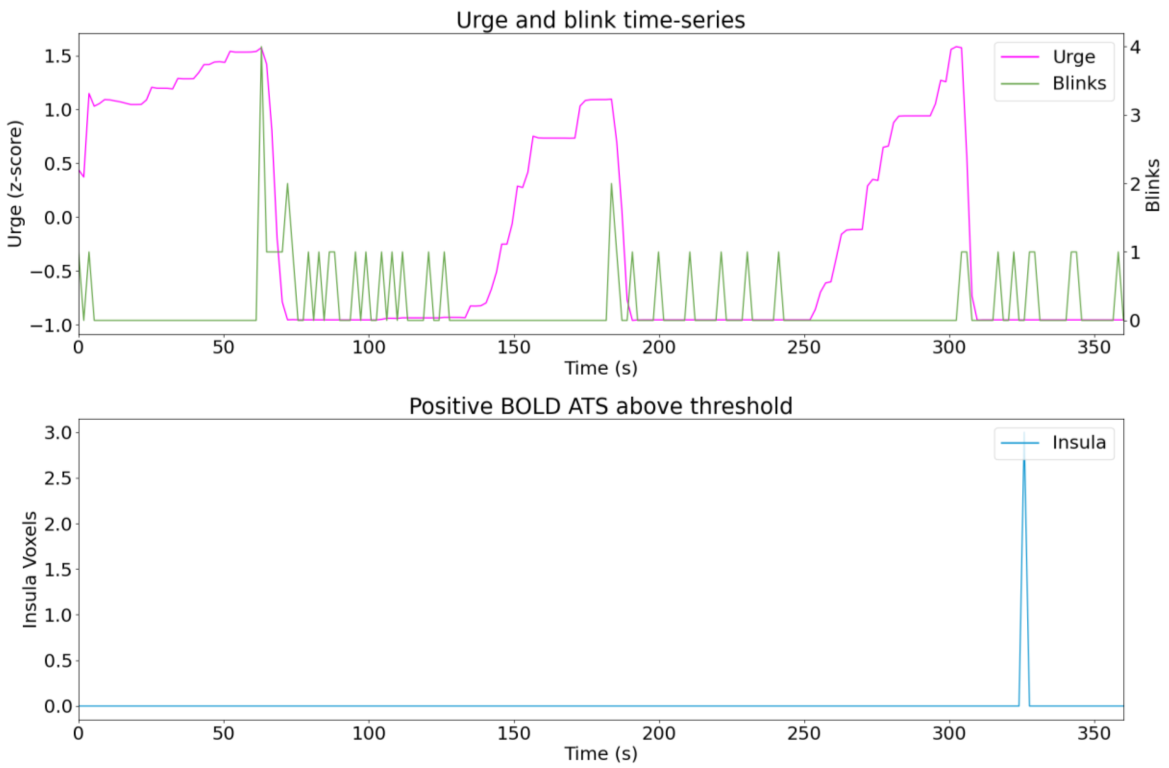

Figure SD.7. *Sub04 run01*

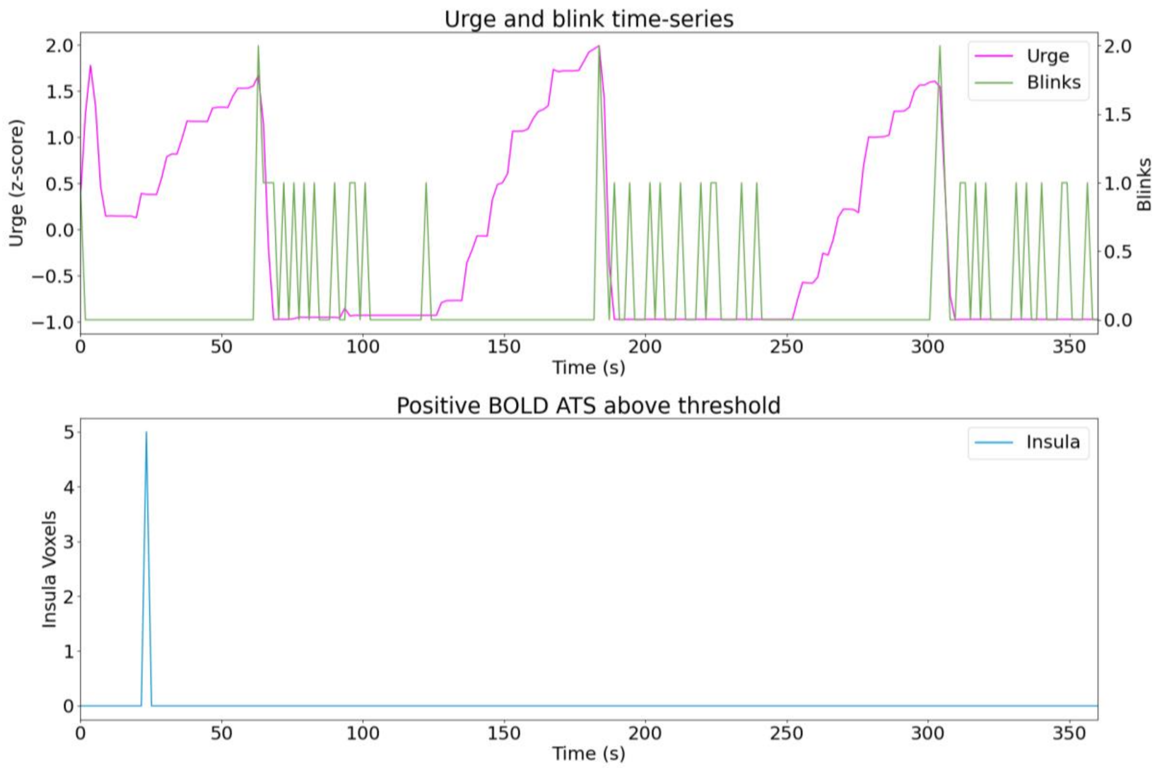

Figure SD.8. *Sub04 run02*

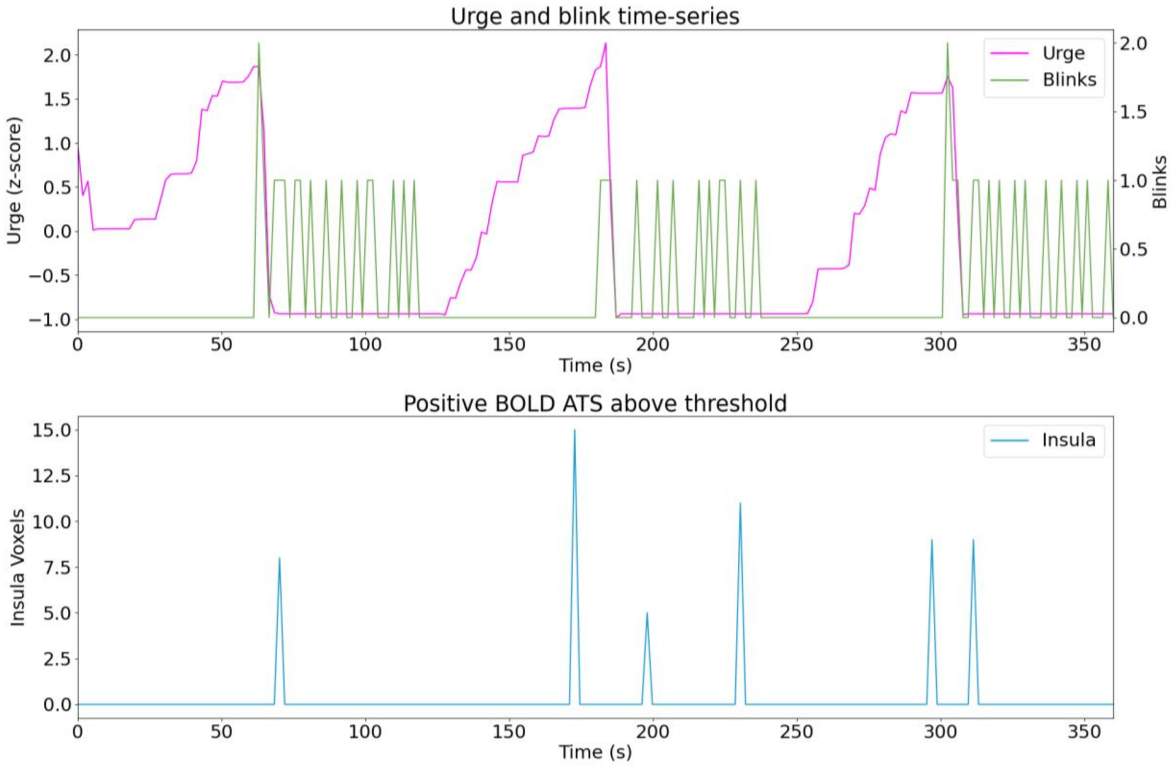

Figure SD.9. *Sub04 run03*

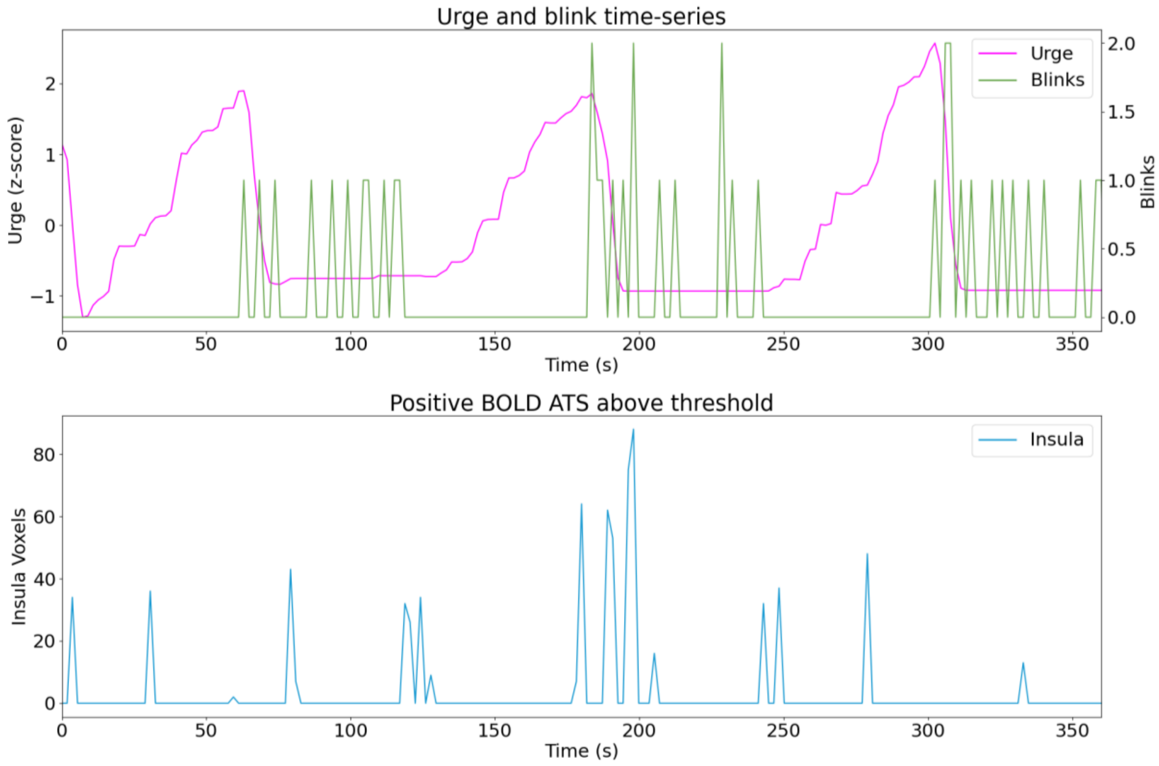

Figure SD.10. *Sub05 run02*

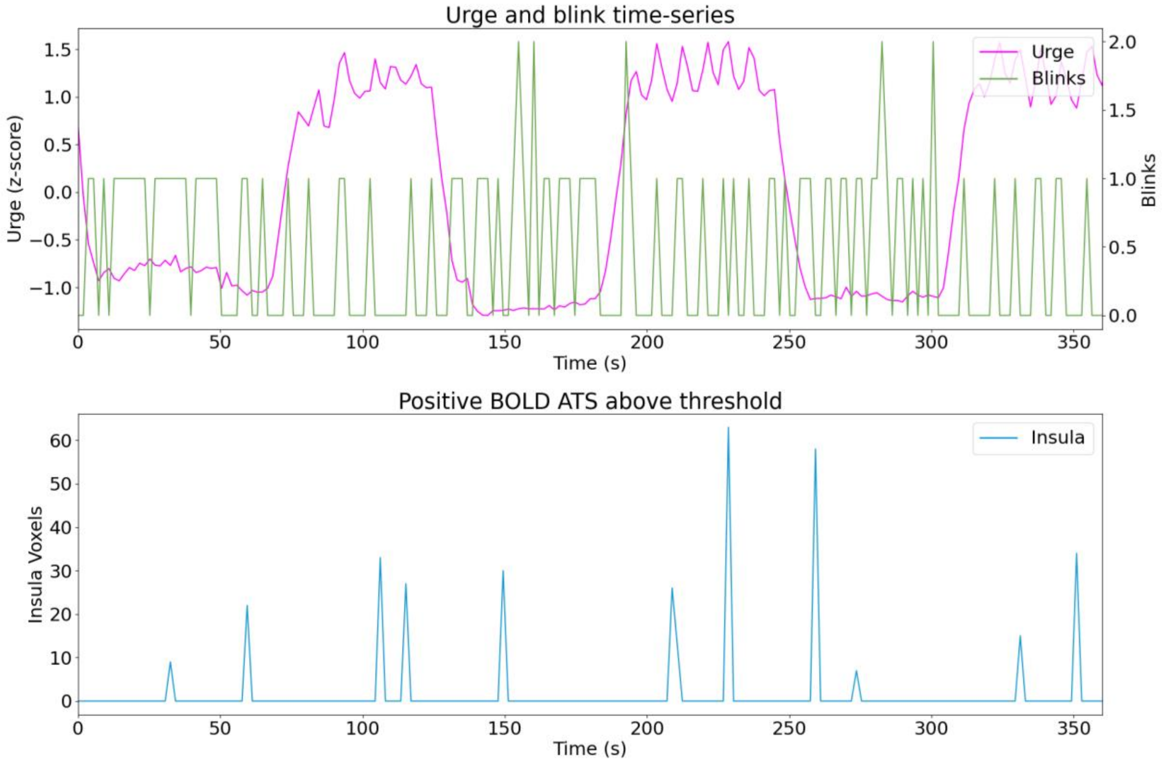

Figure SD.11. *Sub06 run02*

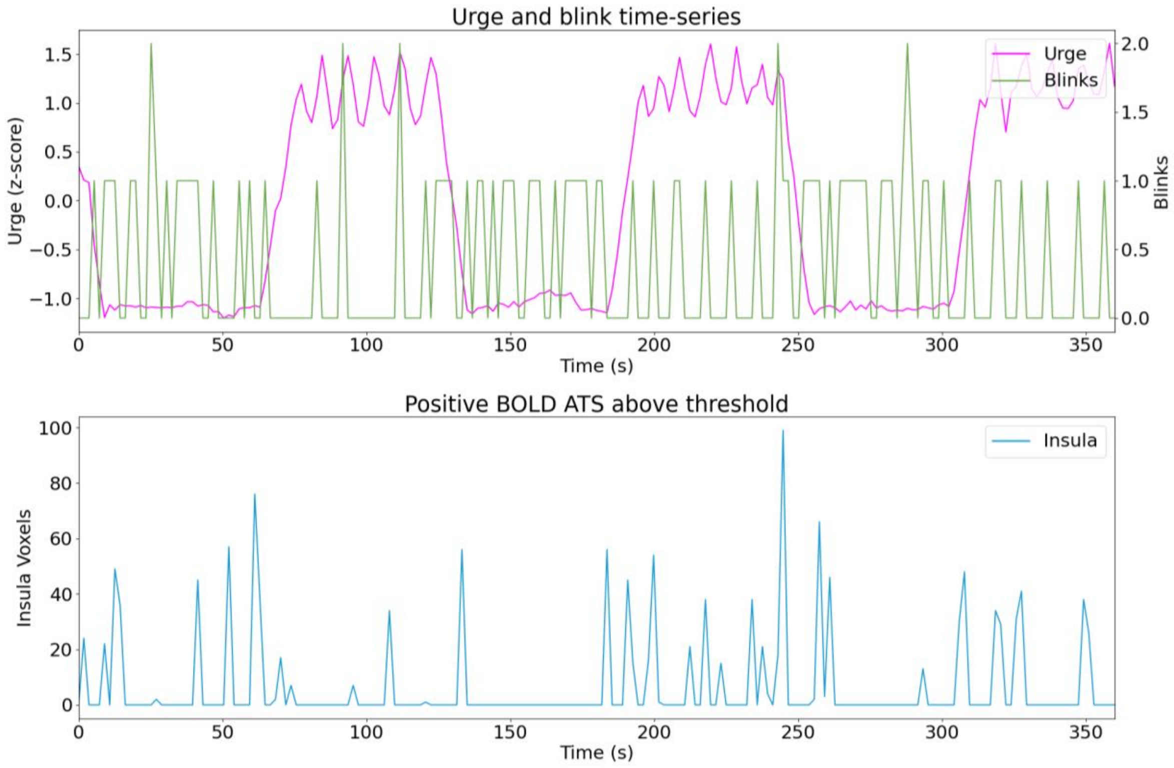

Figure SD.12. *Sub06 run03*

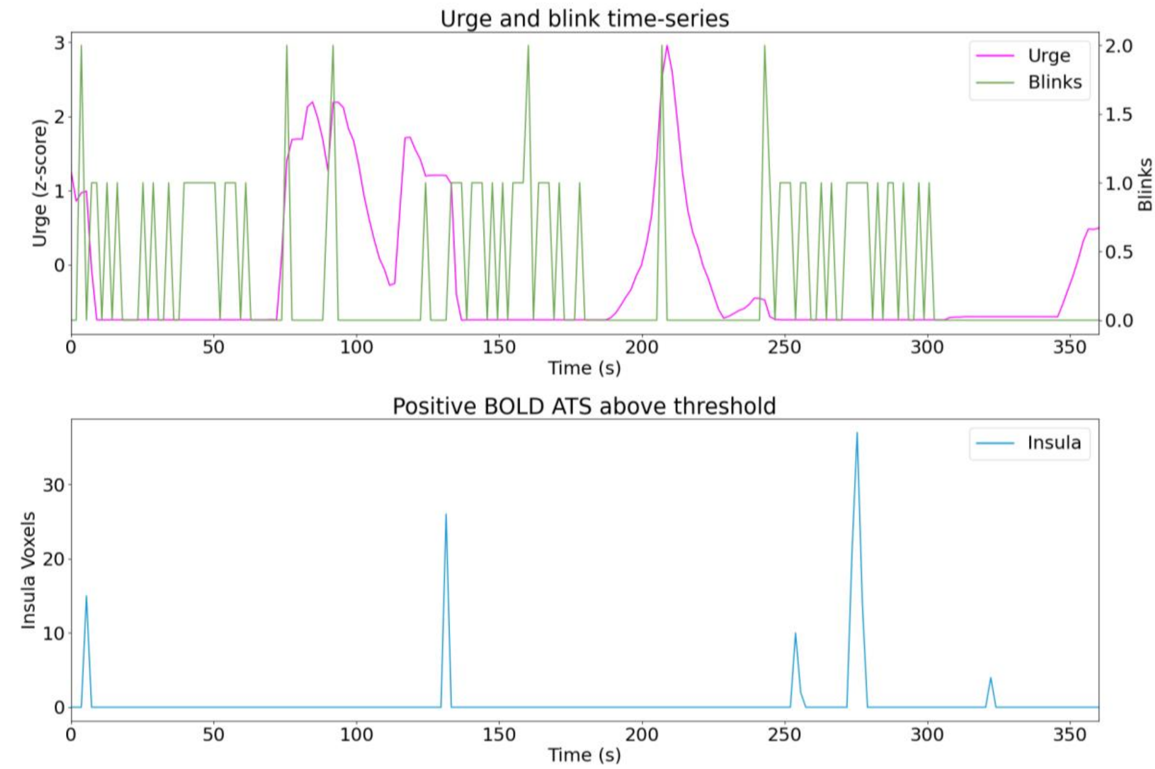

Figure SD.13. *Sub07 run01*

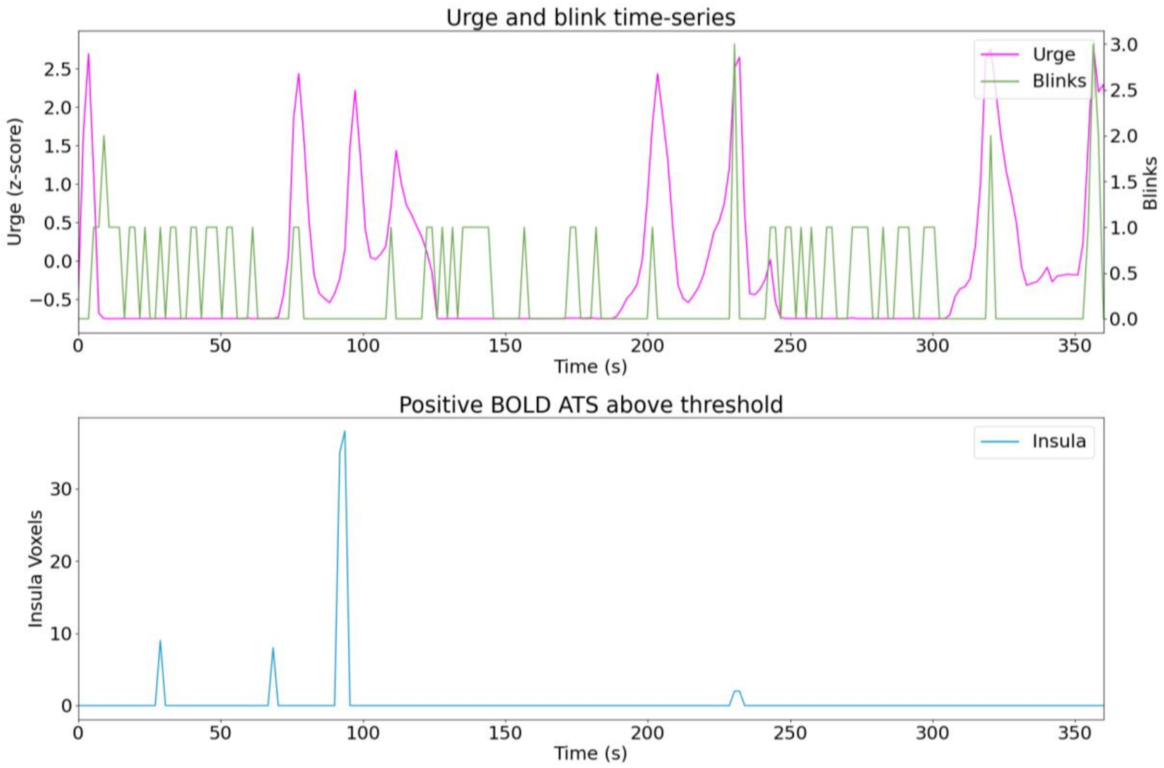

Figure SD.14. *Sub07 run02*

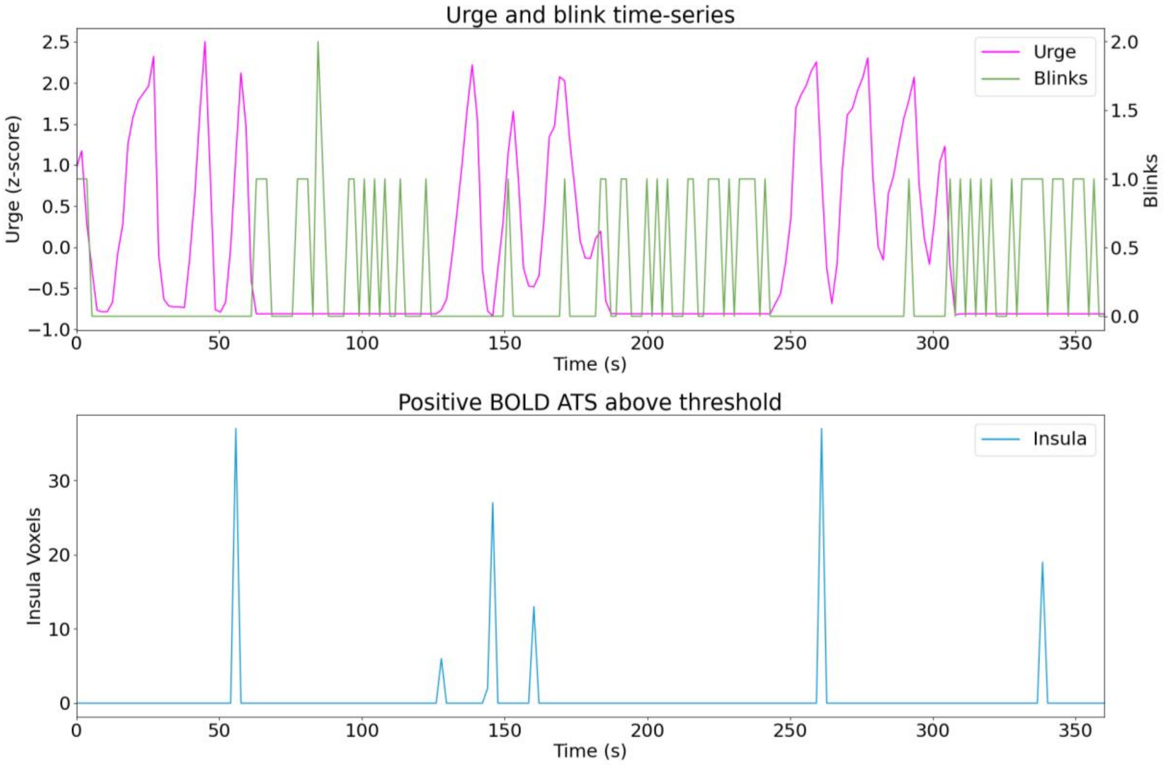

Figure SD.15. *Sub09 run01*

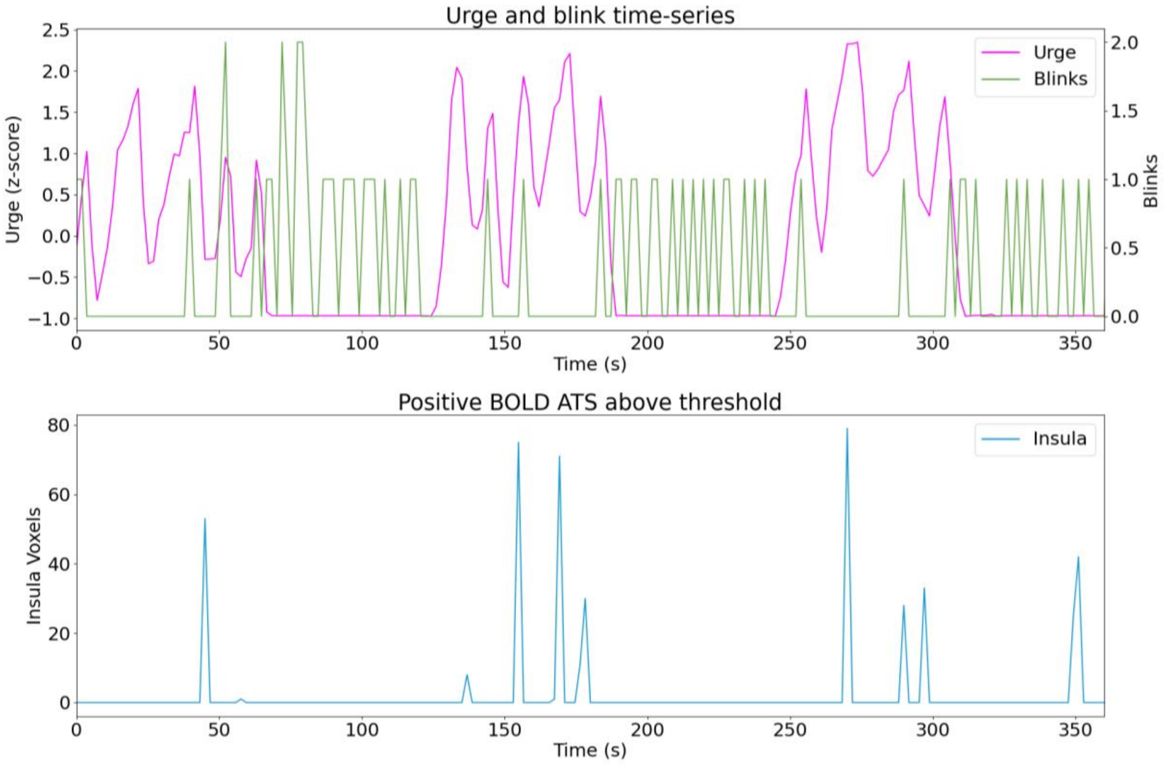

Figure SD.16. *Sub09 run02*

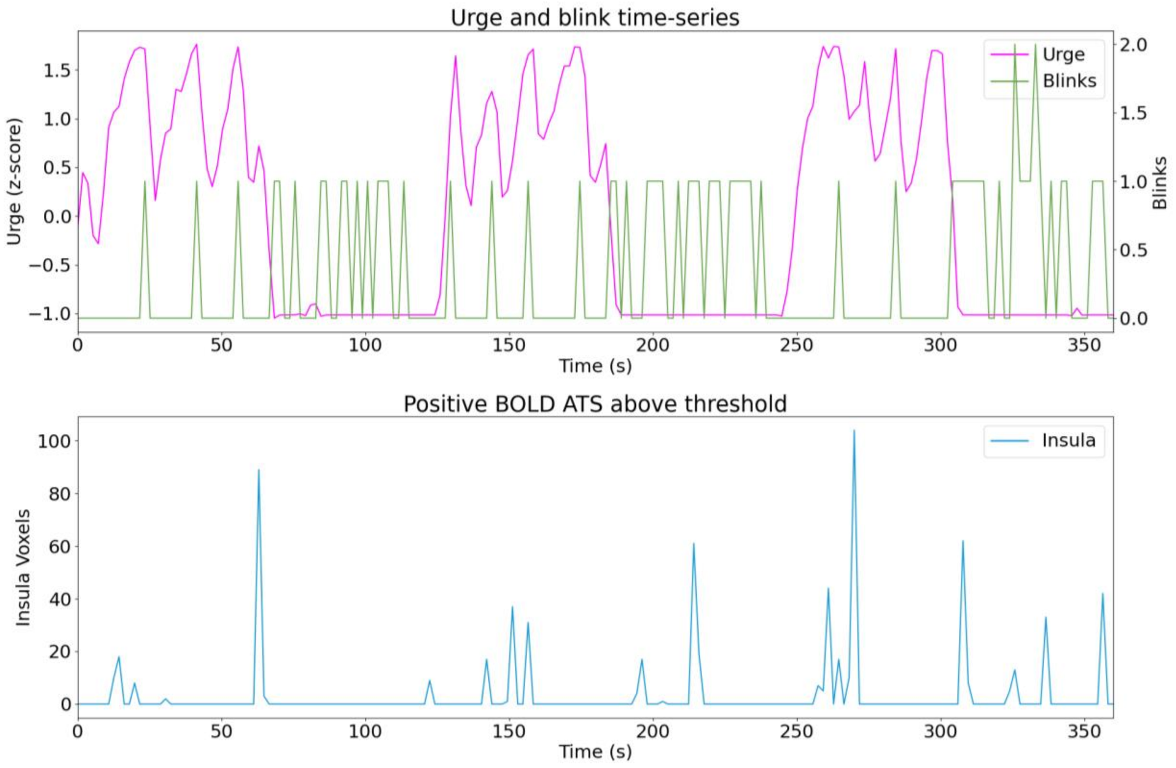

Figure SD.17. *Sub09 run03*

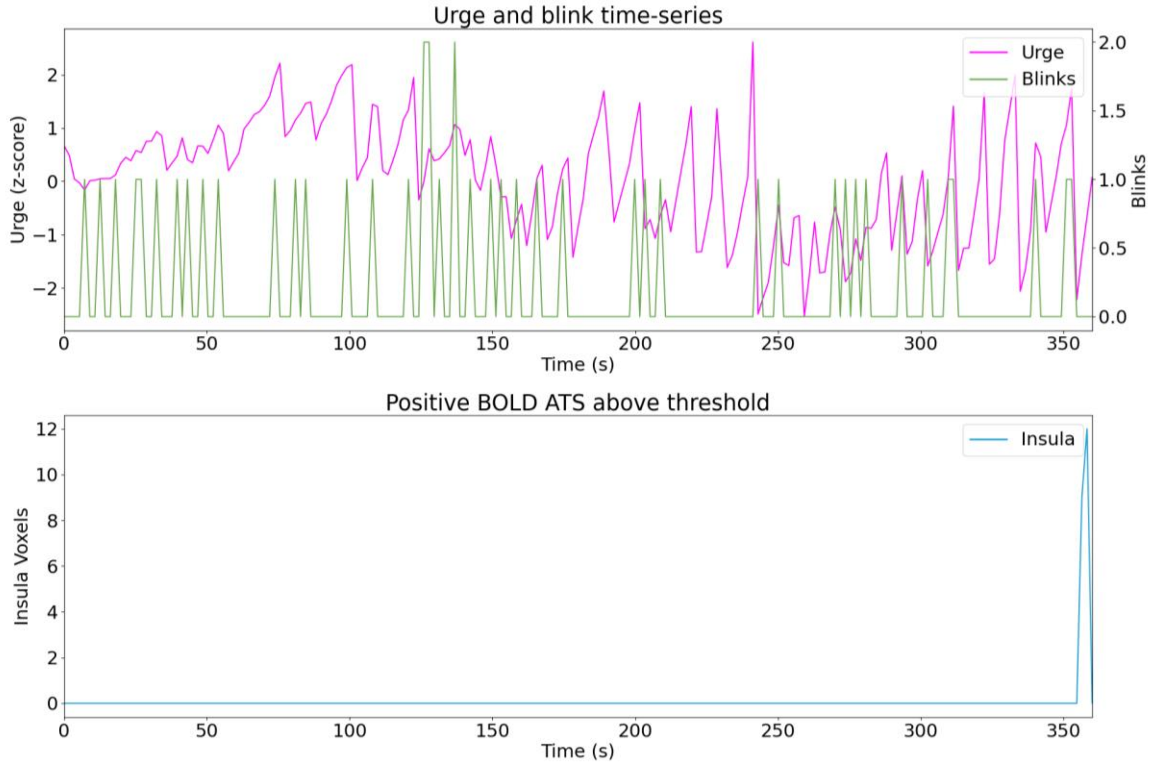

Figure SD.18. *Sub10 run01*

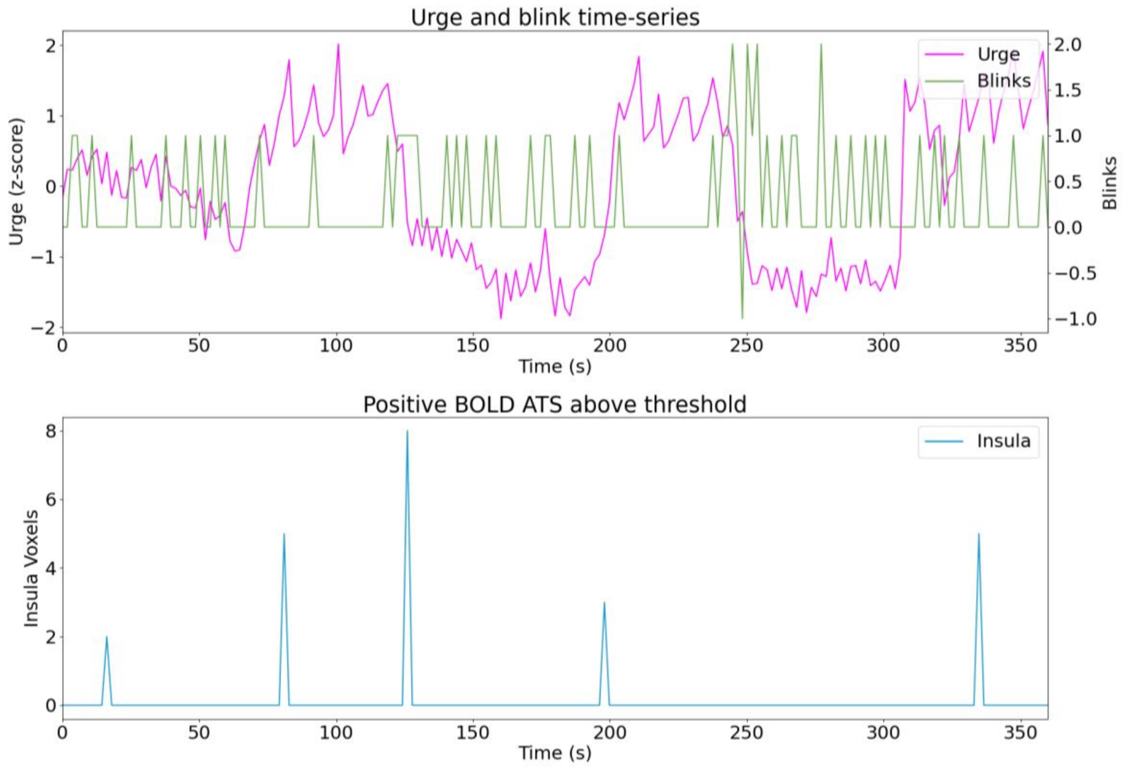

Figure SD.19. *Sub10 run02*

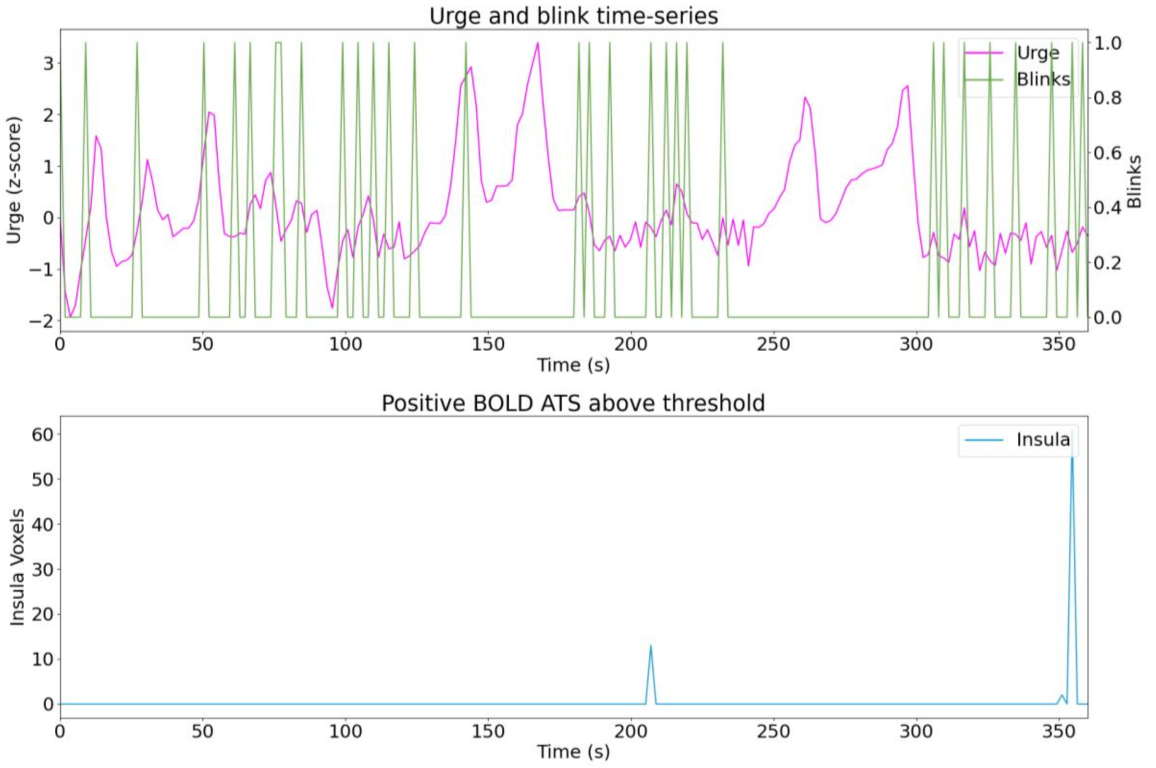

Figure SD.20. *Sub11 run01*

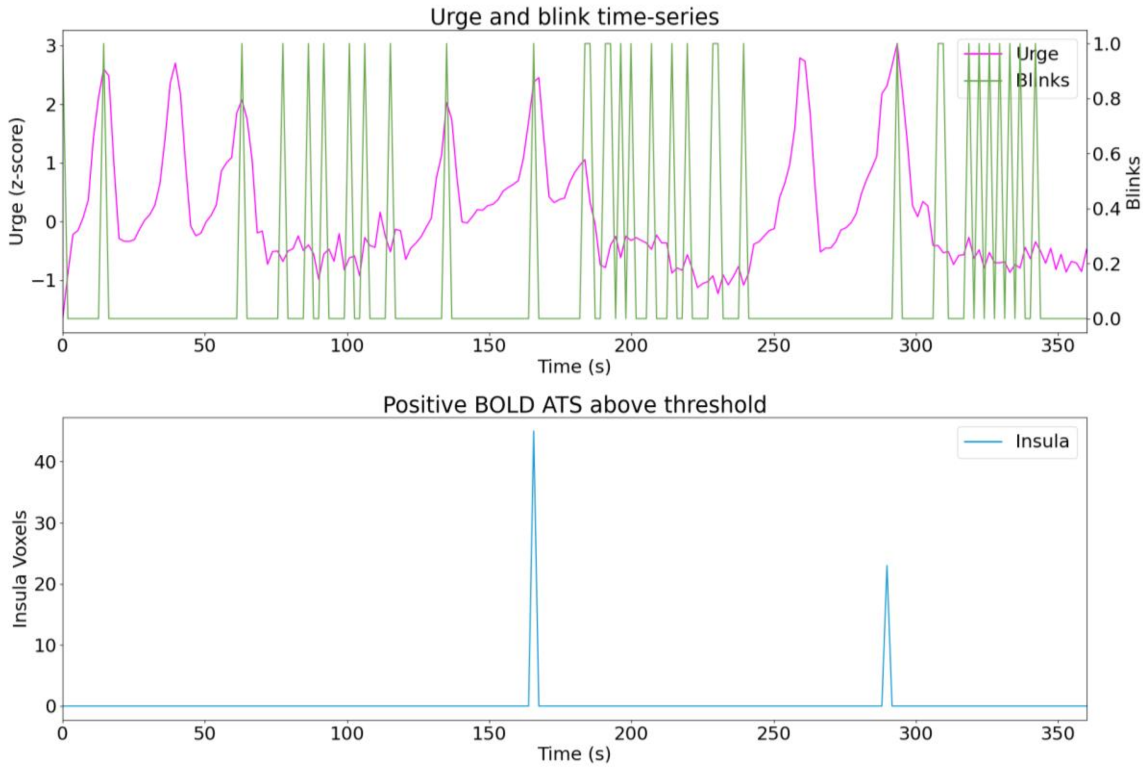

Figure SD.21. *Sub11 run02*

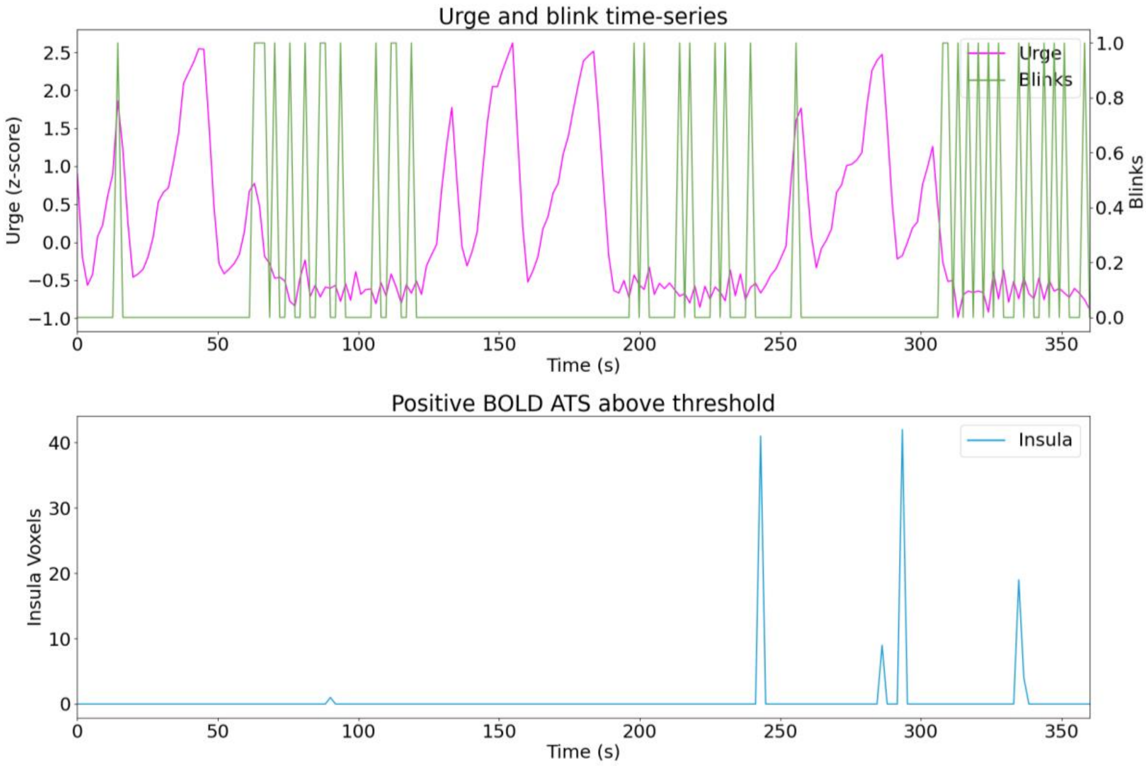

Figure SD.22. *Sub11 run03*

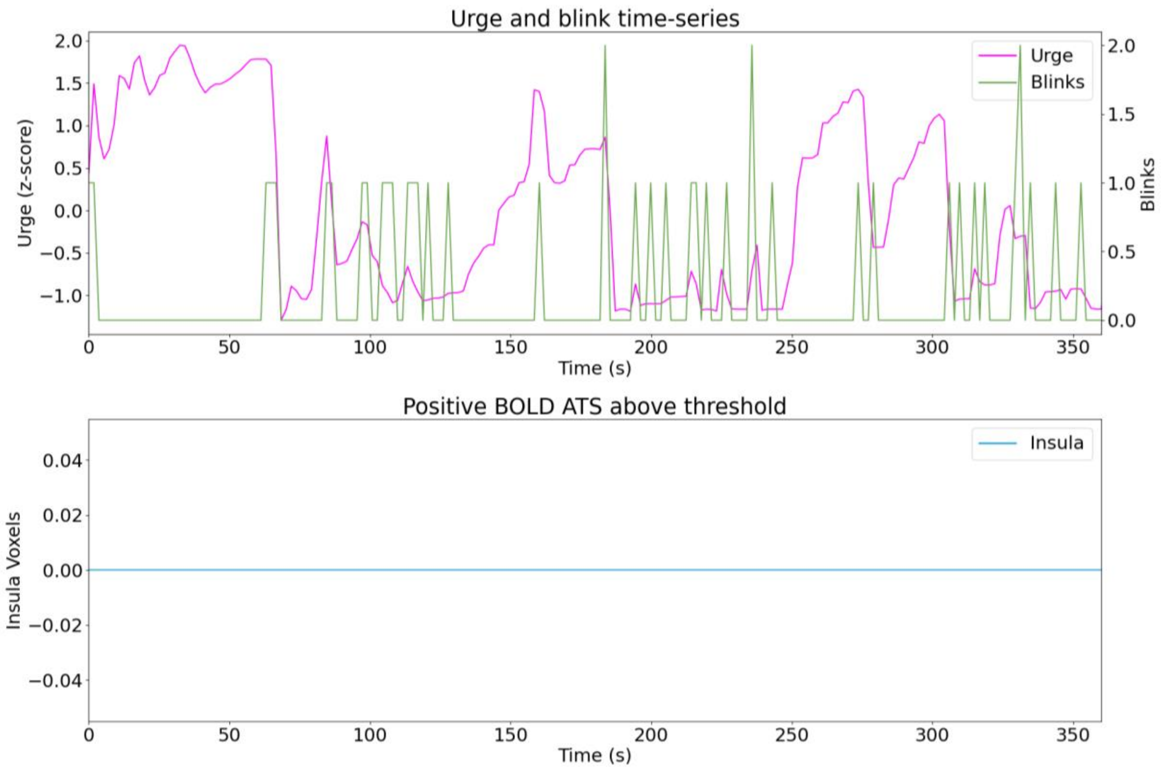

Figure SD.23. *Sub12 run01*

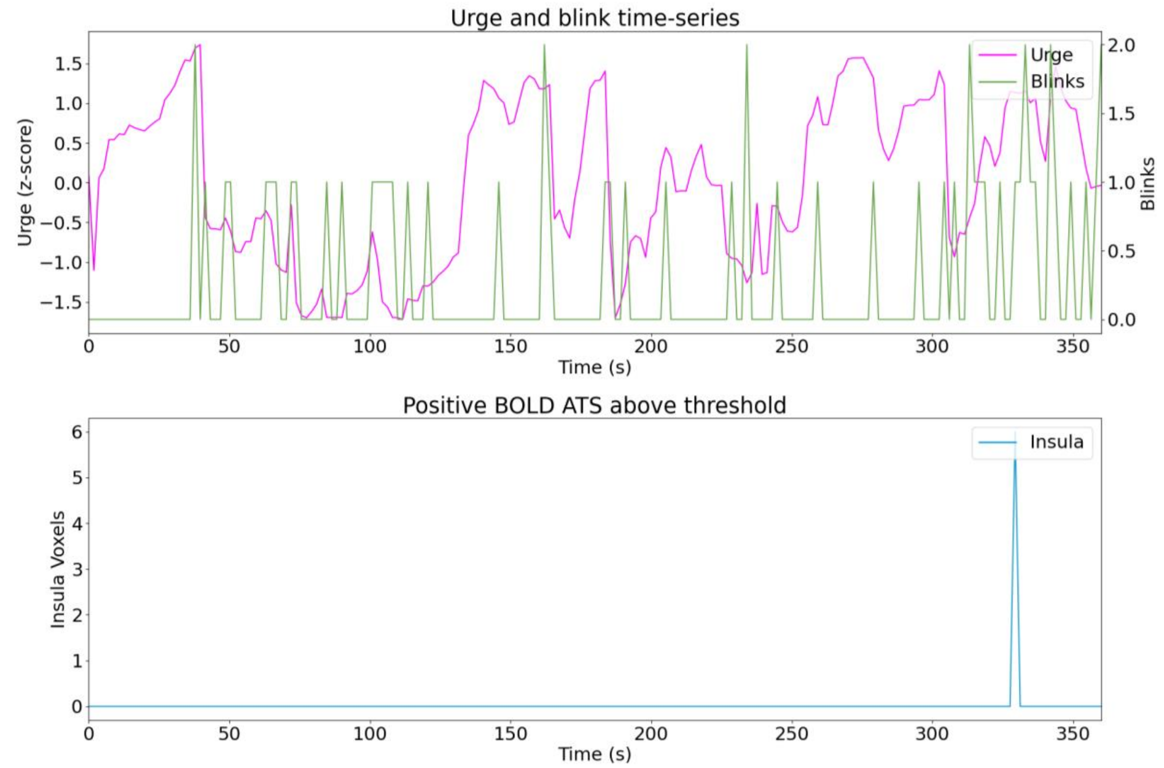

Figure SD.24. *Sub12 run02*

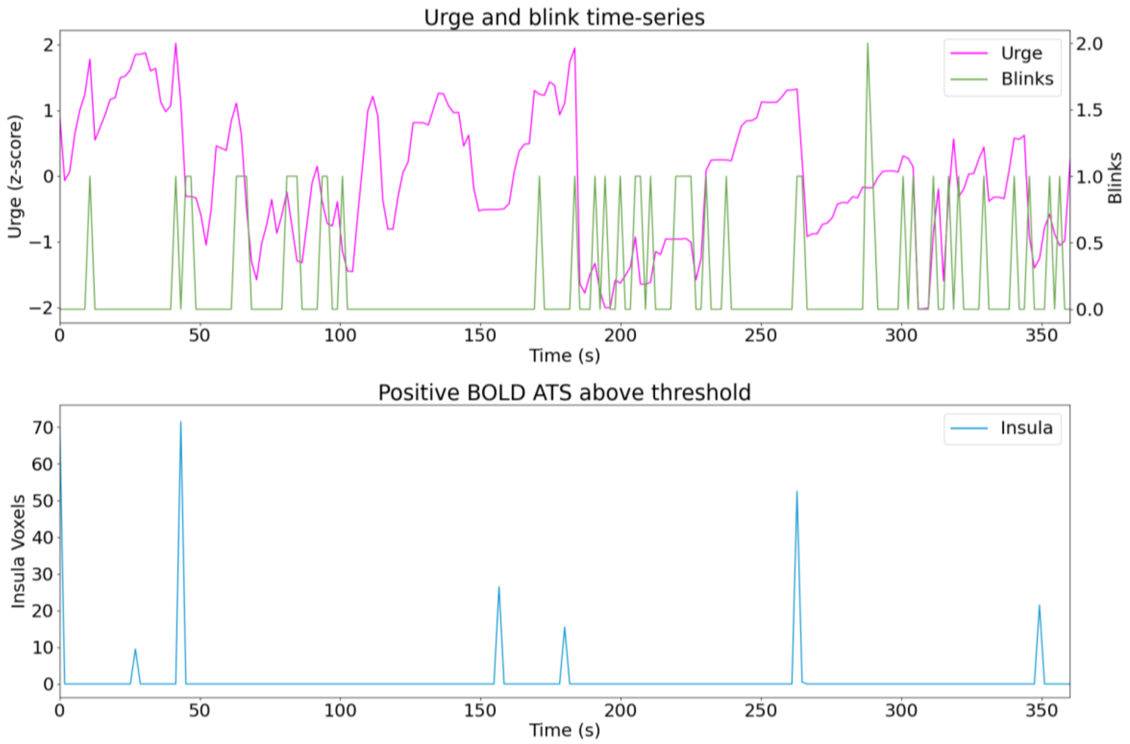

Figure SD.25. *Sub12 run03*

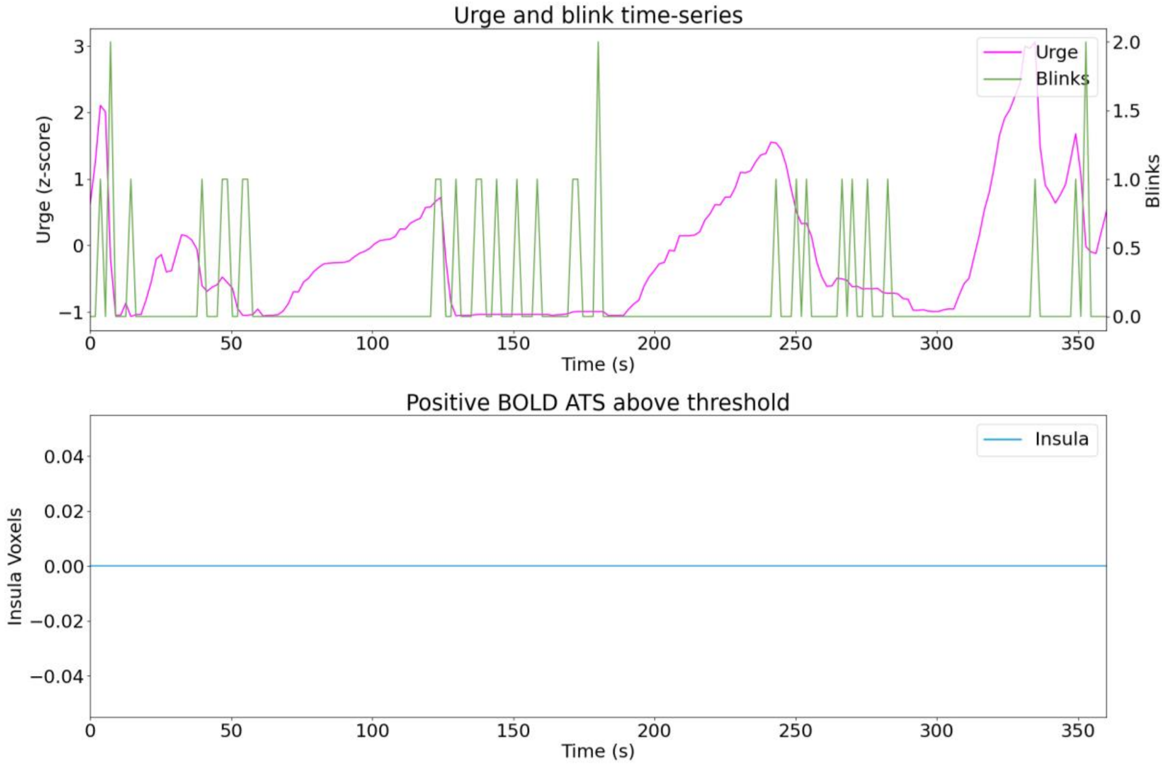

Figure SD.26. *Sub13 run01*

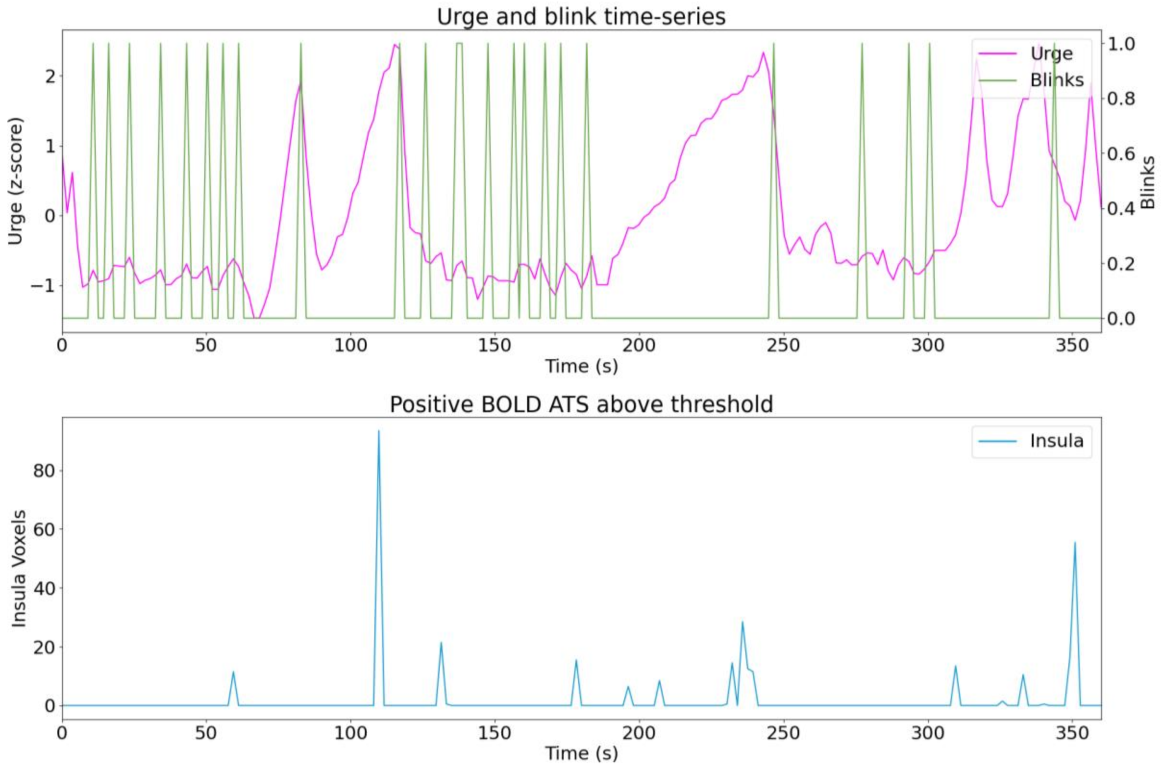

Figure SD.27. *Sub13 run02*

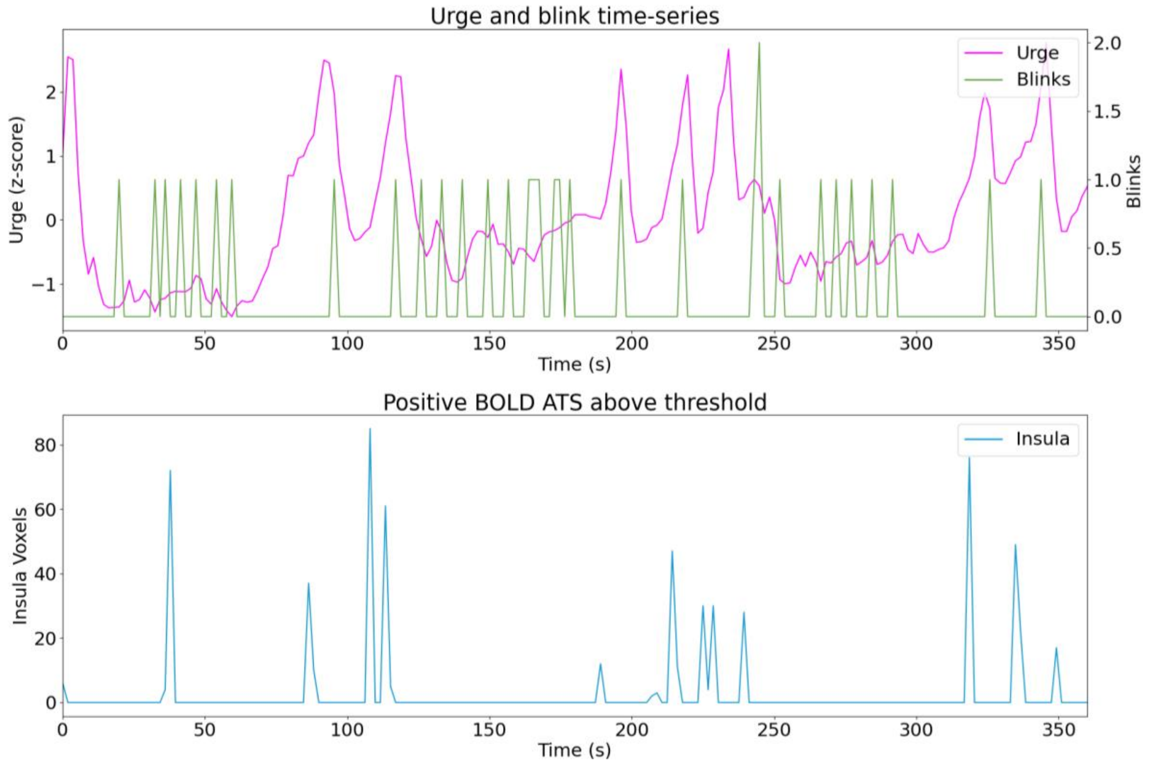

Figure SD.28. *Sub13 run03*

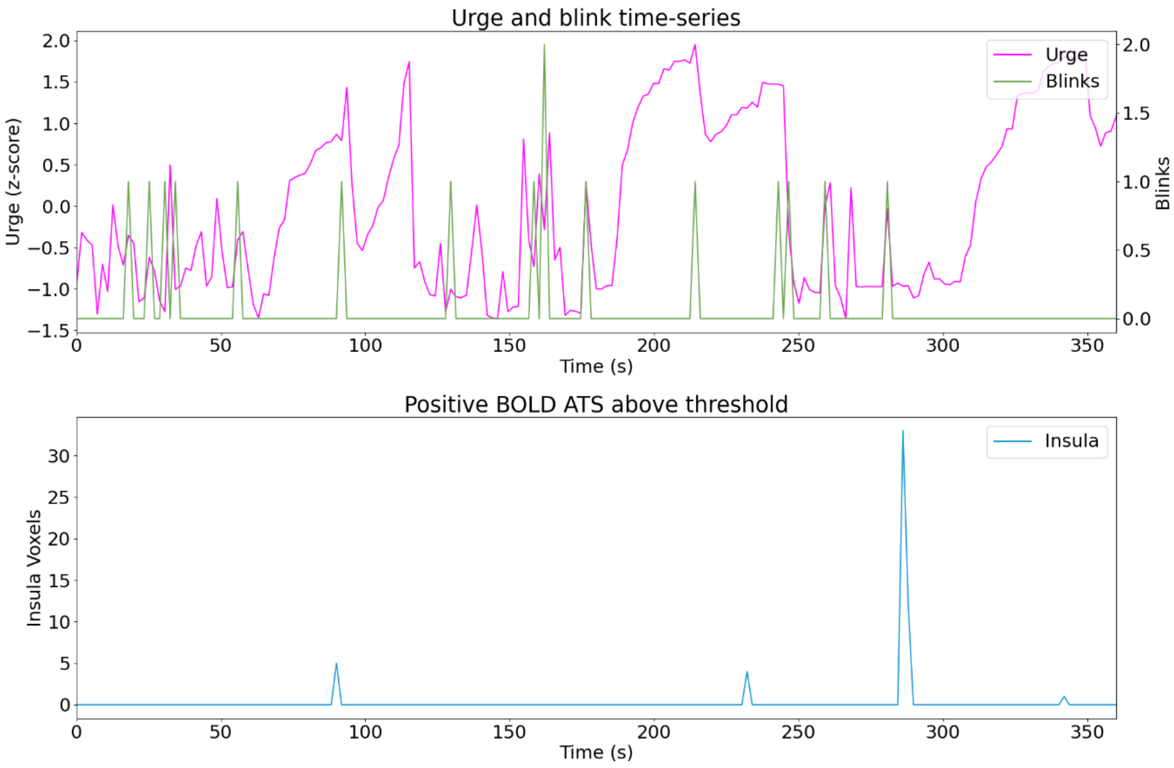

Figure SD.29. *Sub14 run01*

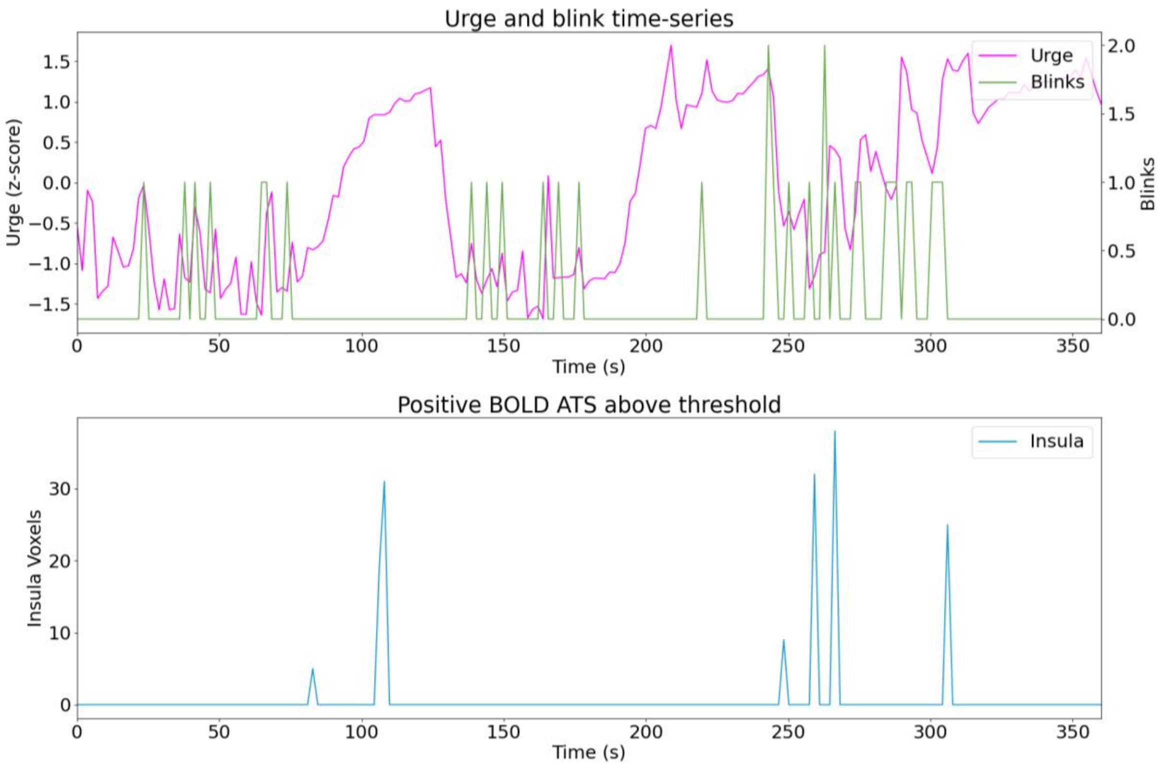

Figure SD.30. *Sub14 run02*

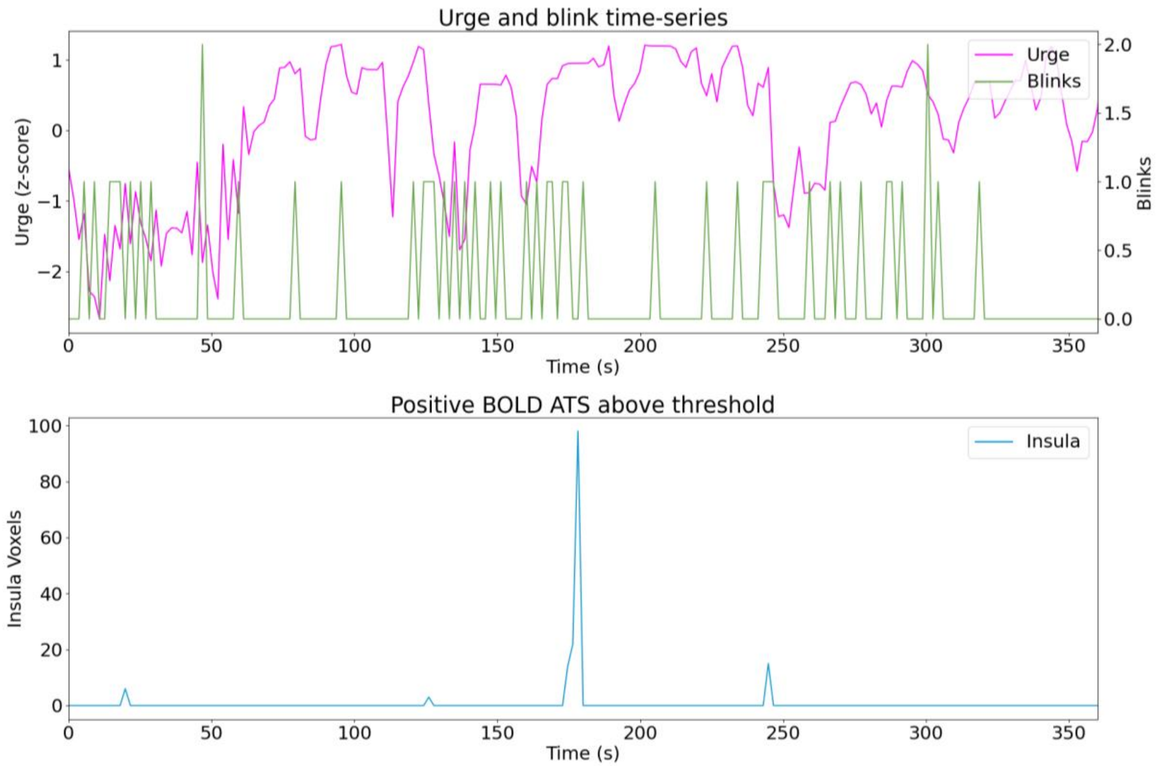

Figure SD.31. *Sub14 run03*

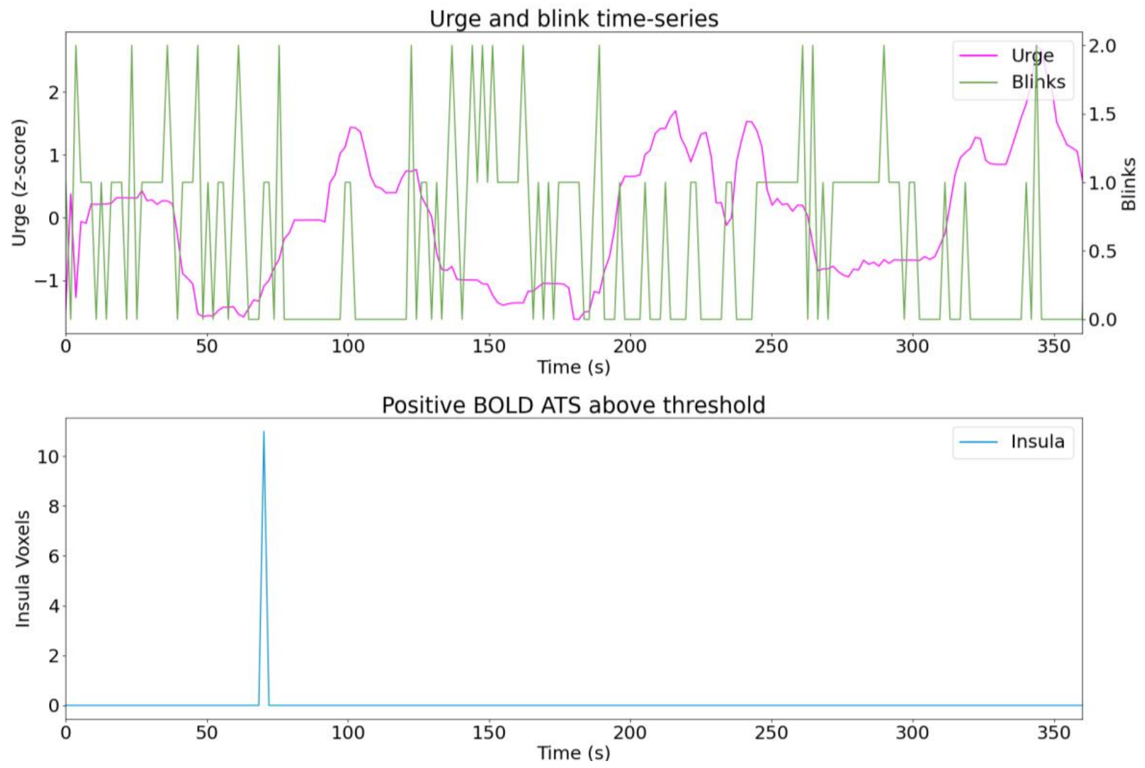

Figure SD.32. *Sub15 run01*

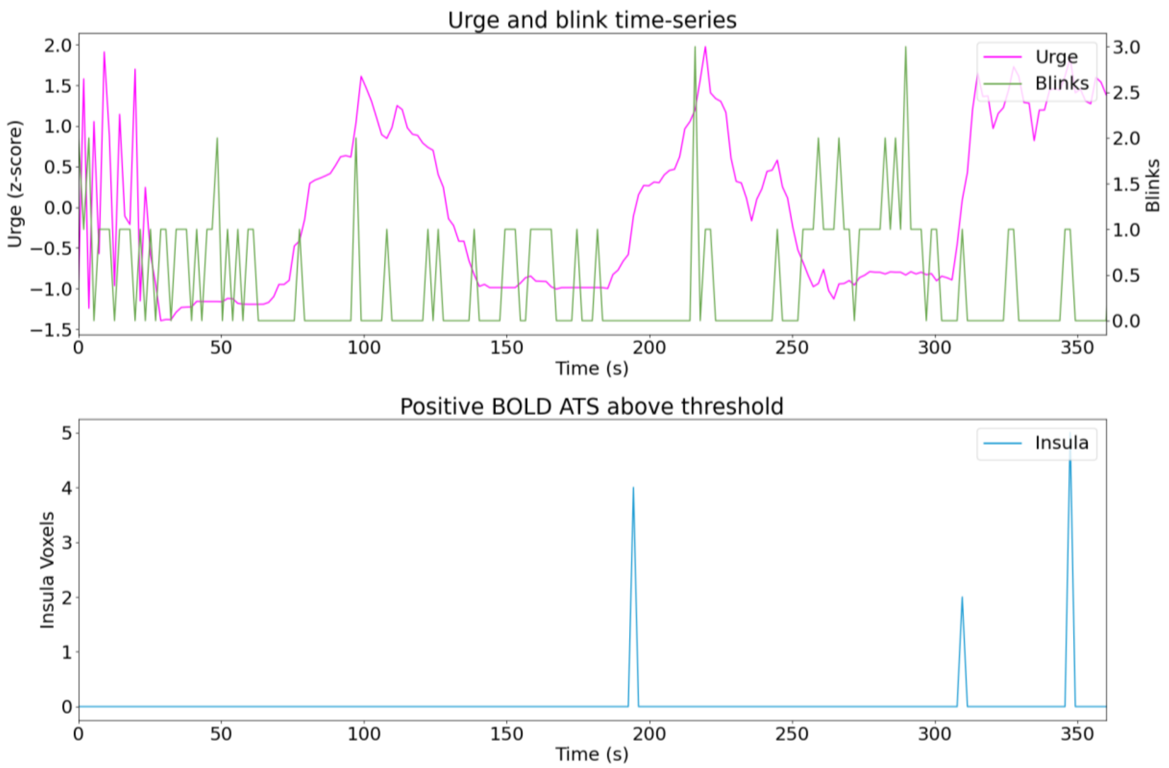

Figure SD.33. *Sub15 run02*

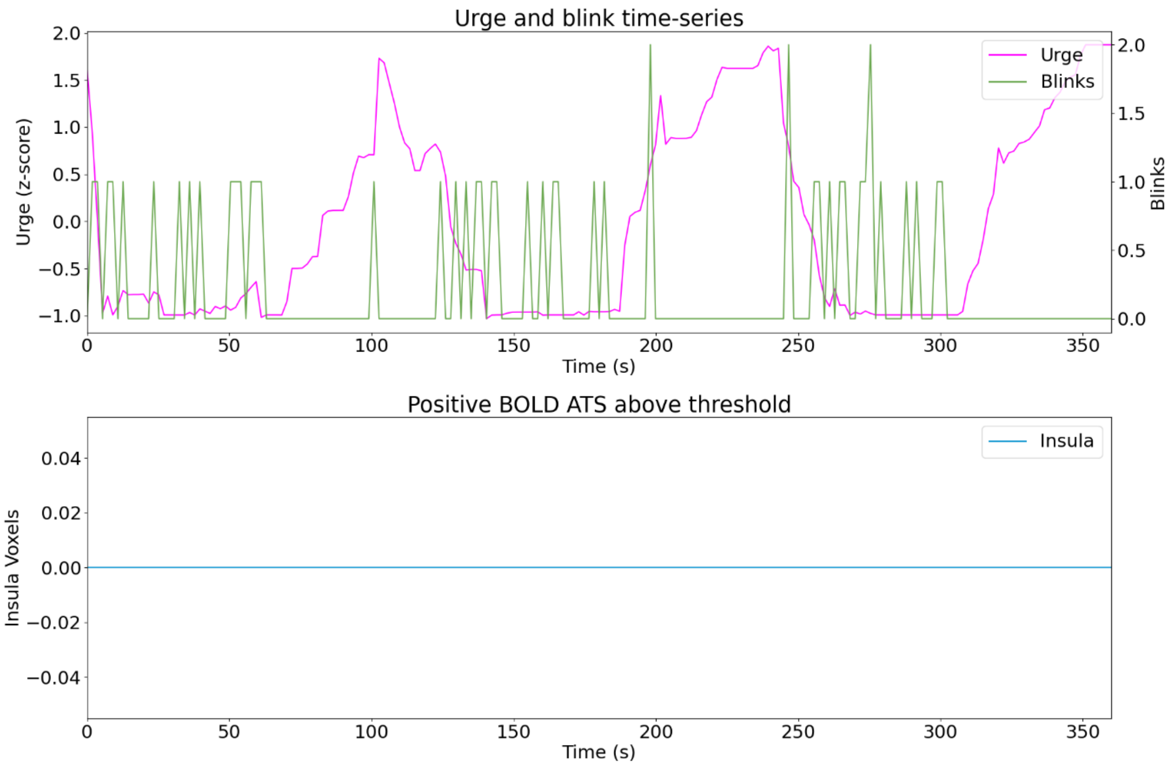

Figure SD.34. *Sub15 run03*

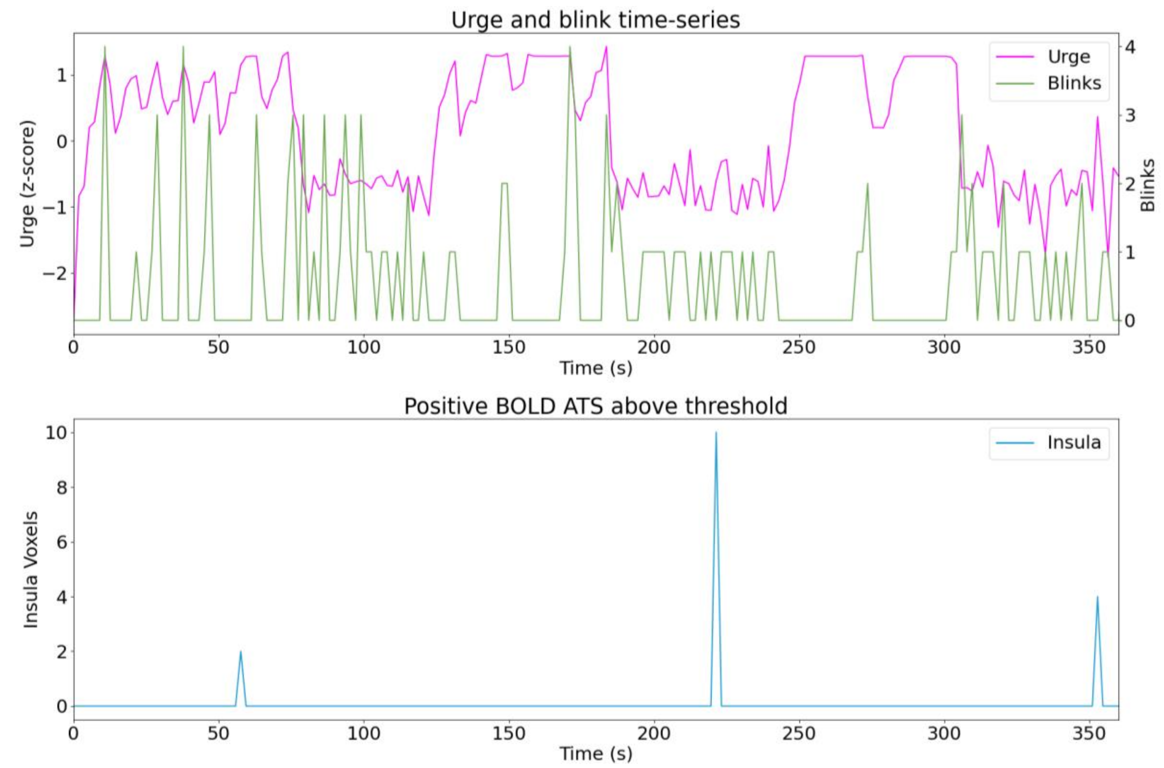

Figure SD.35. *Sub16 run01*

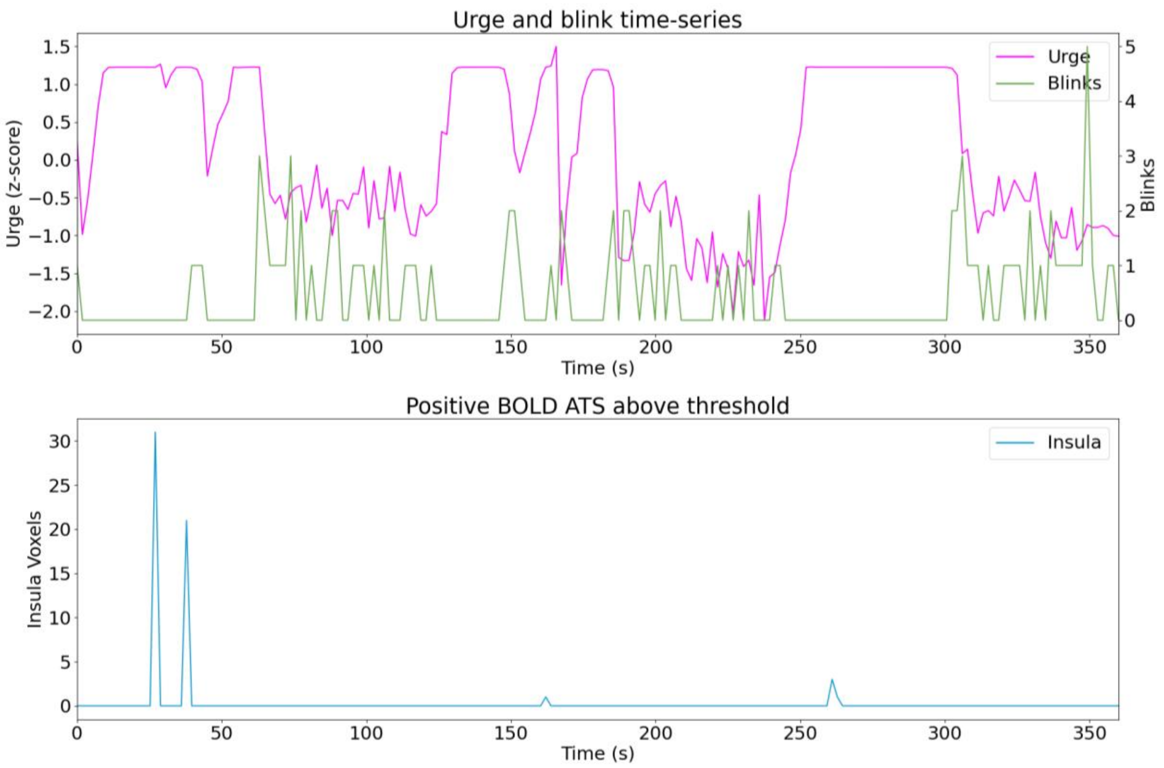

Figure SD.36. *Sub16 run02*

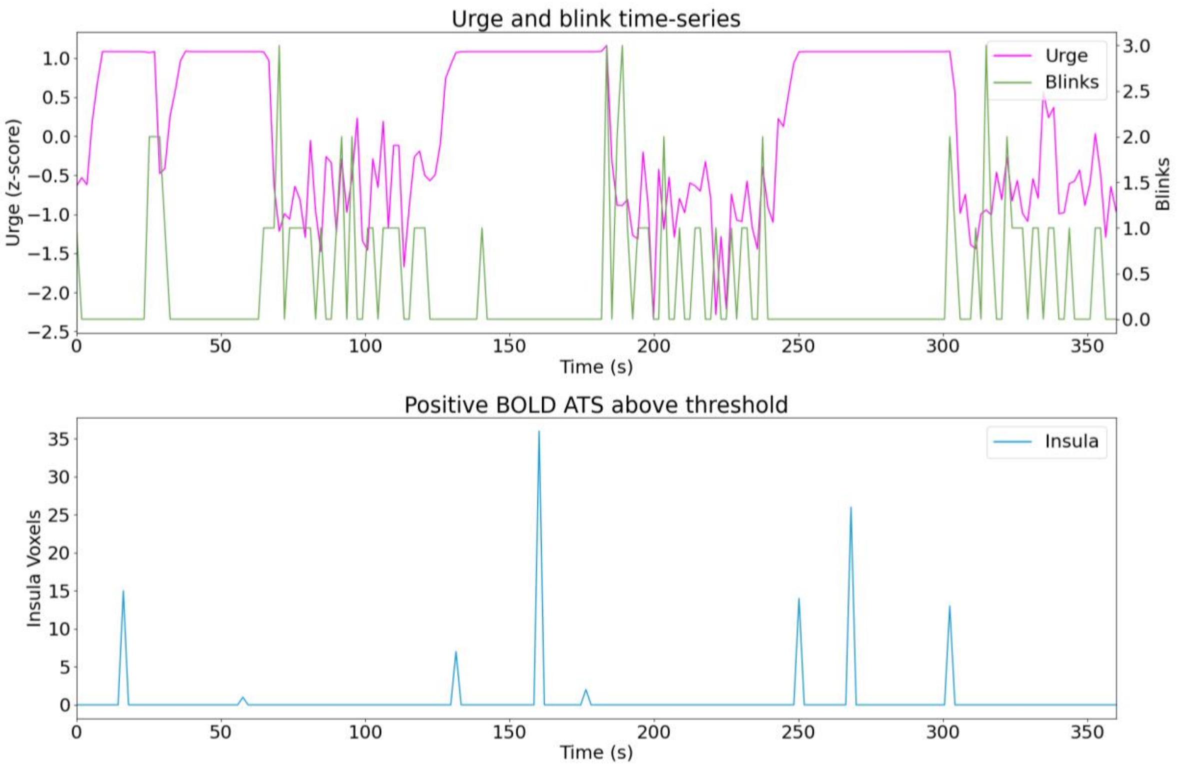

Figure SD.37. *Sub16 run03*

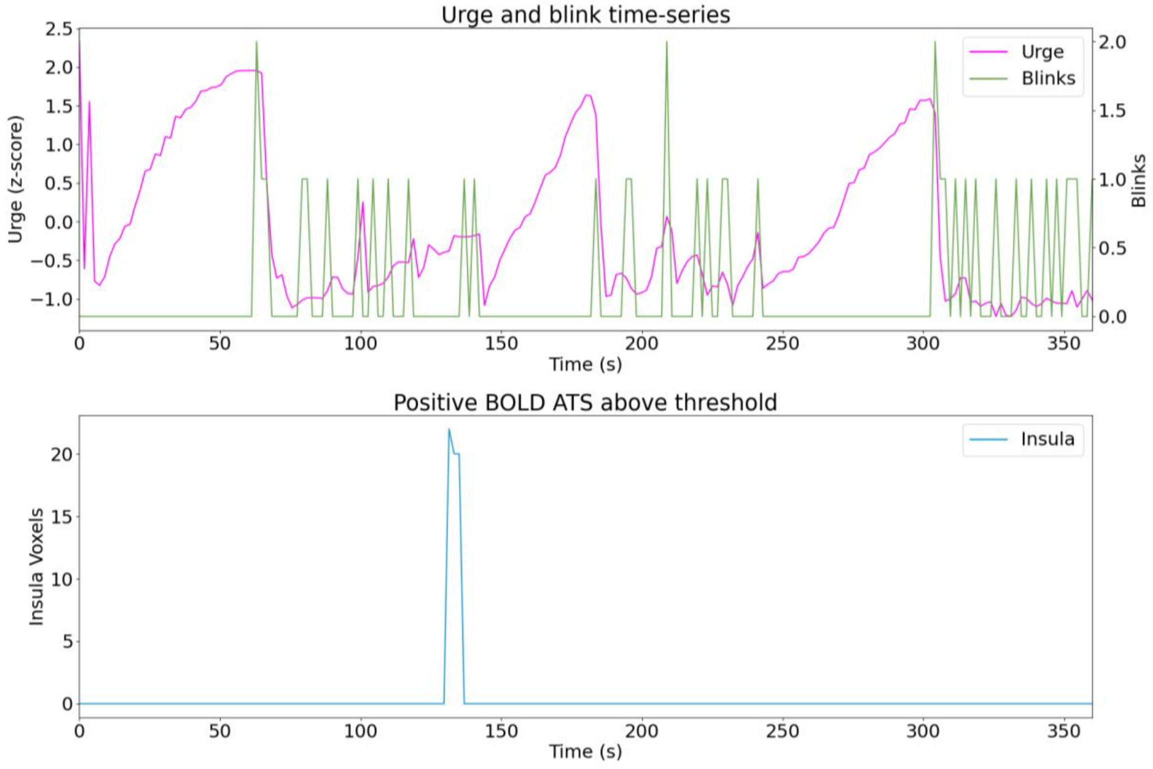

Figure SD.38. *Sub17 run01*

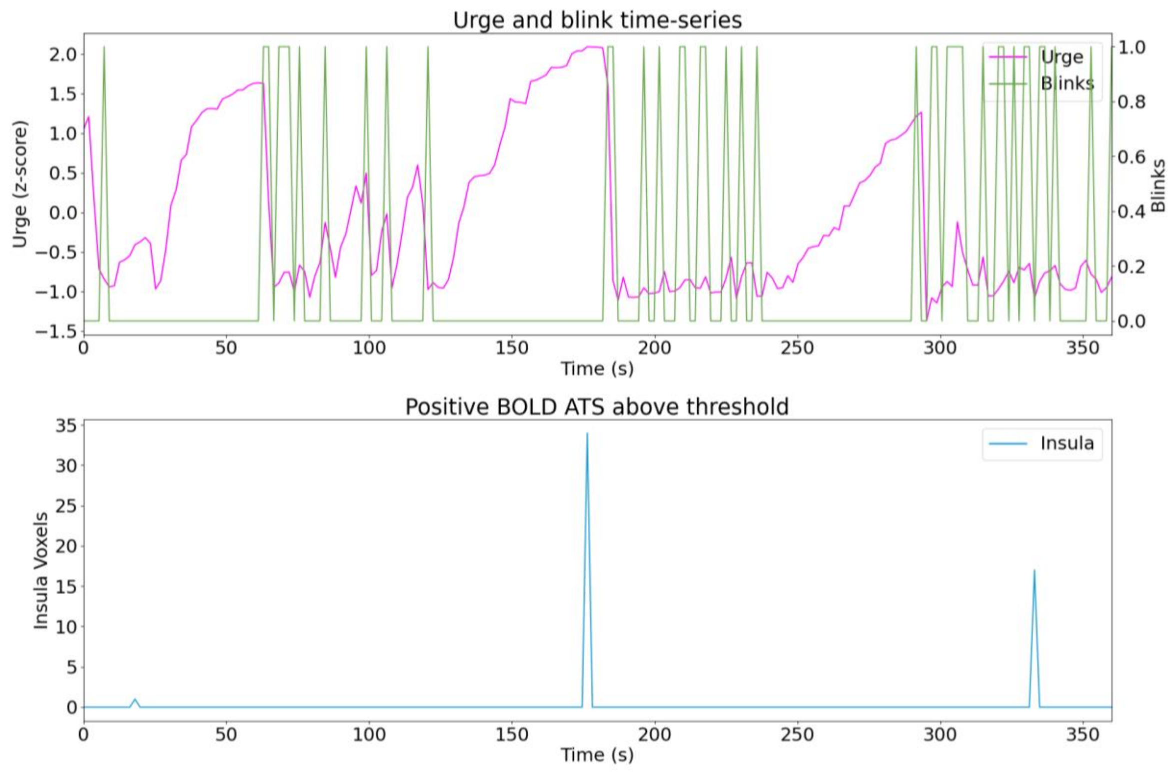

Figure SD.39. *Sub17 run02*

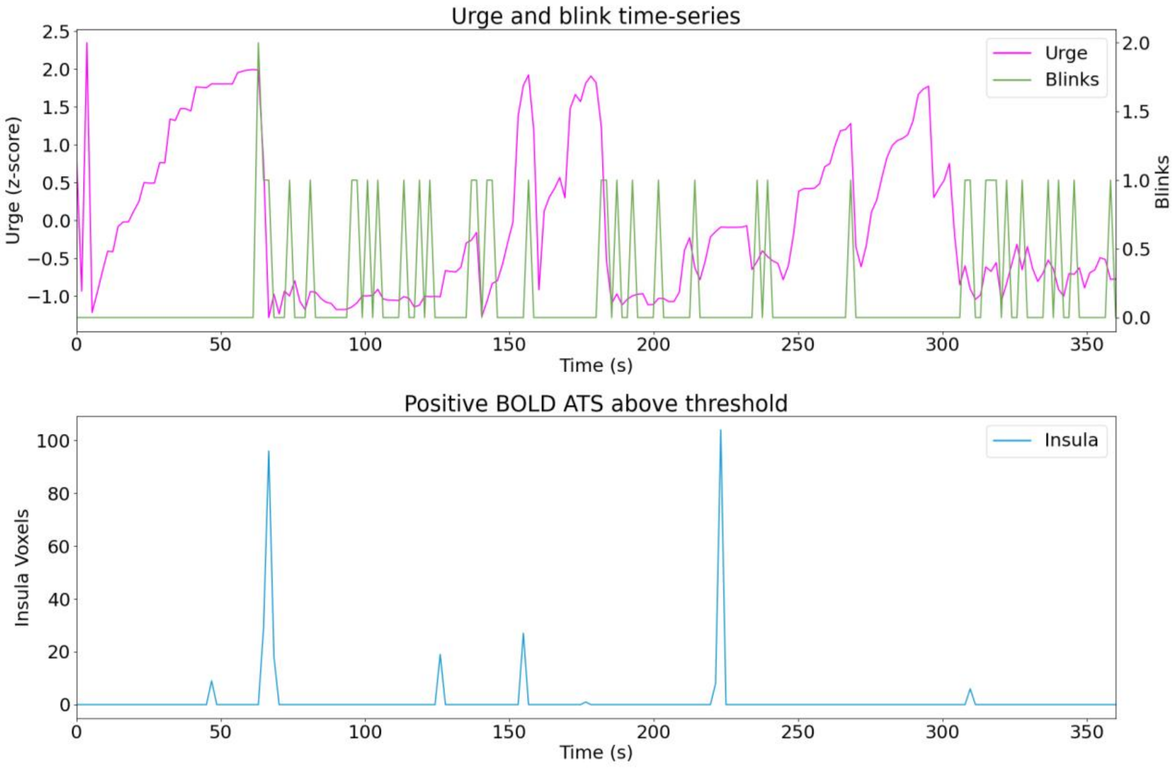

Figure SD.40. *Sub17 run03*

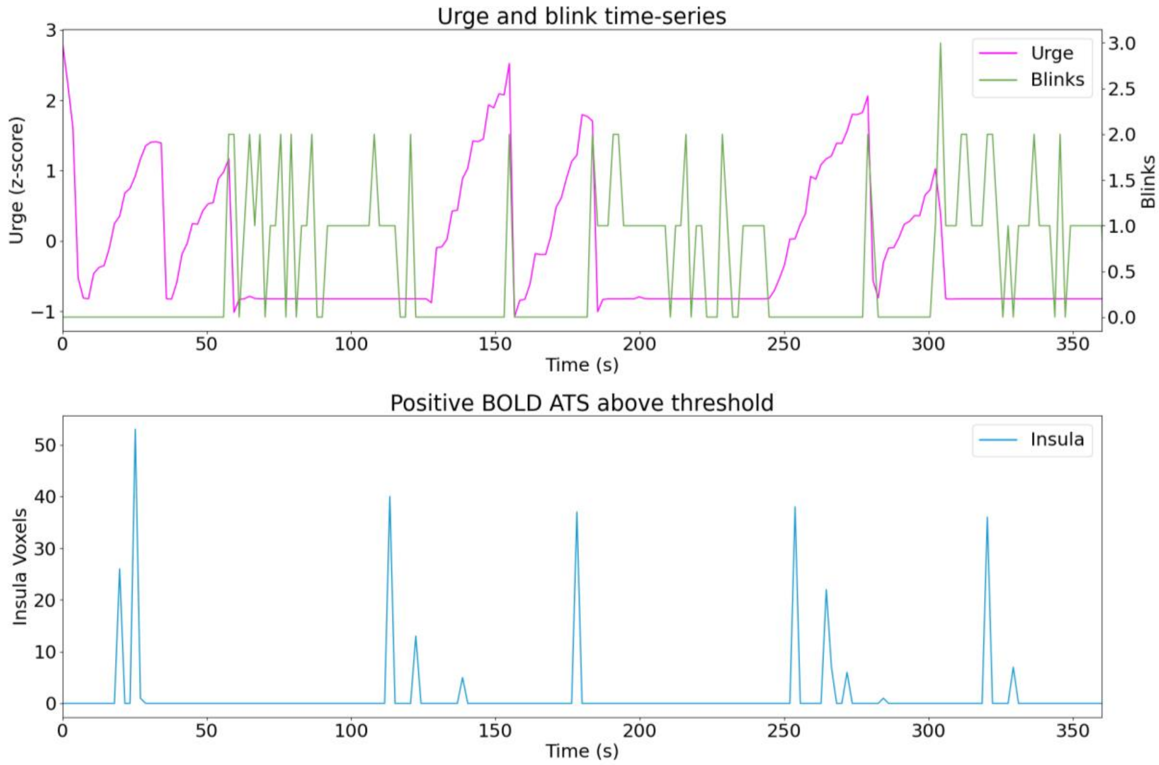

Figure SD.41. *Sub18 run01*

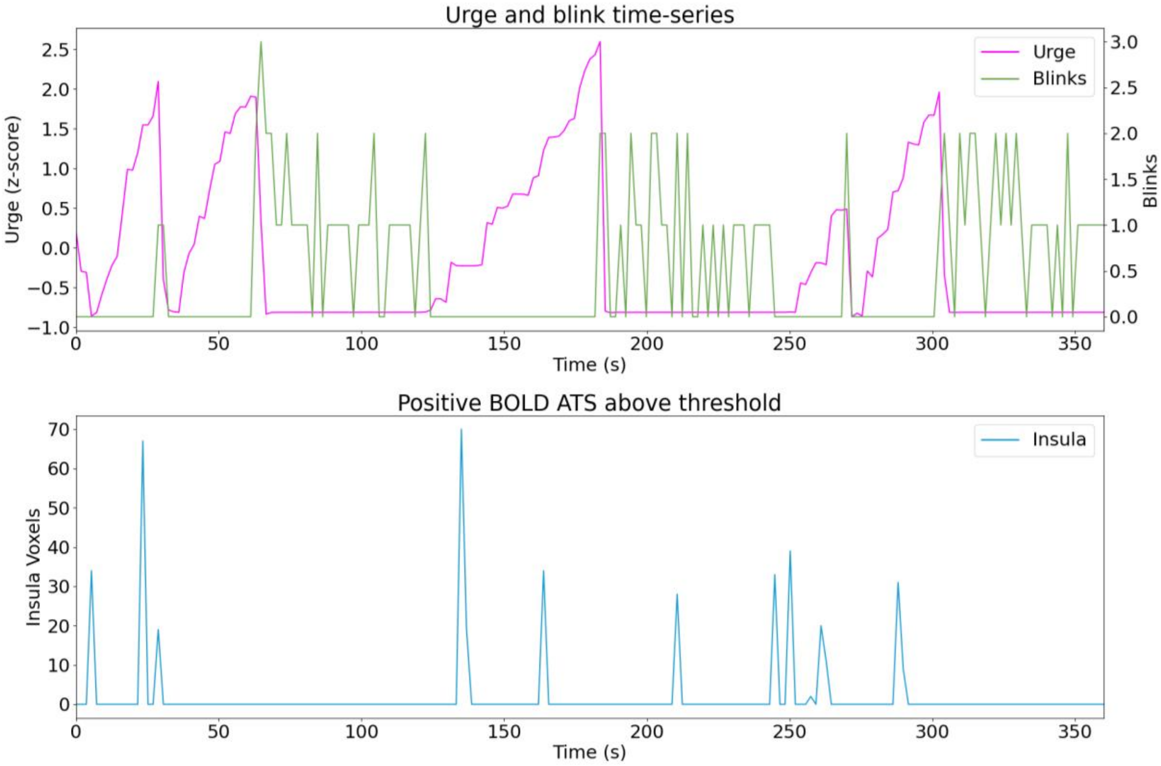

Figure SD.42. *Sub18 run02*

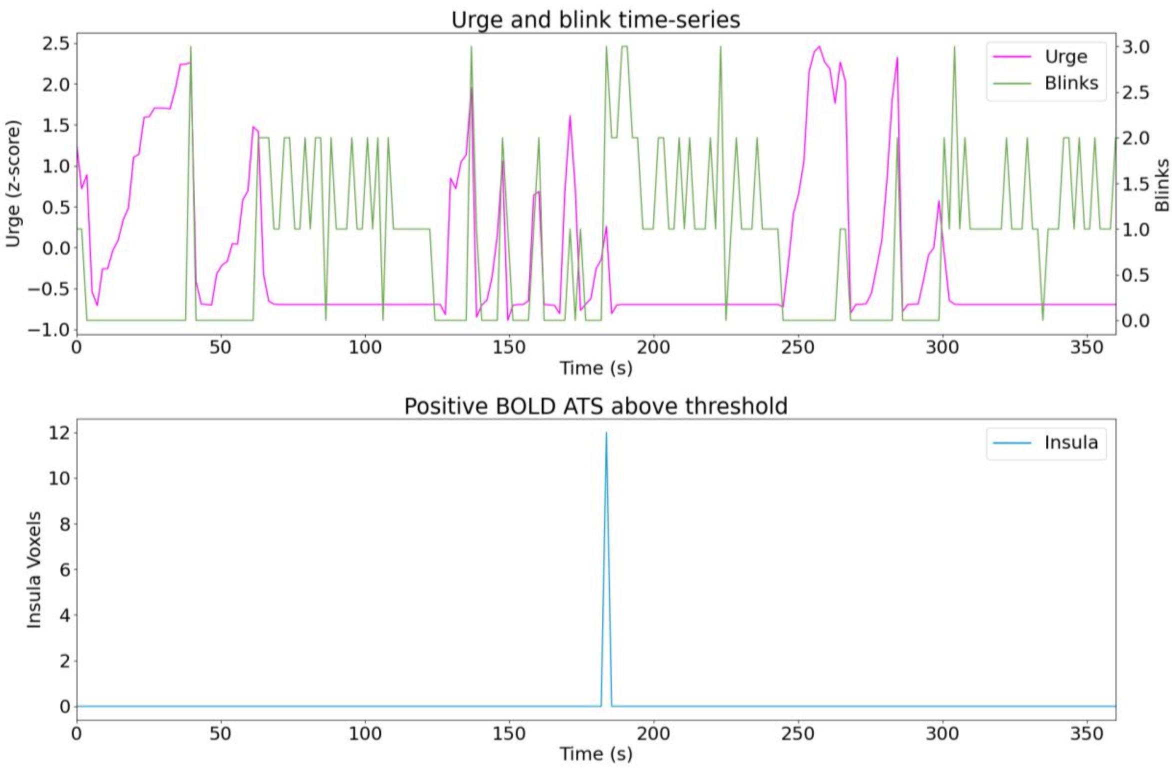

Figure SD.43. *Sub18 run03*

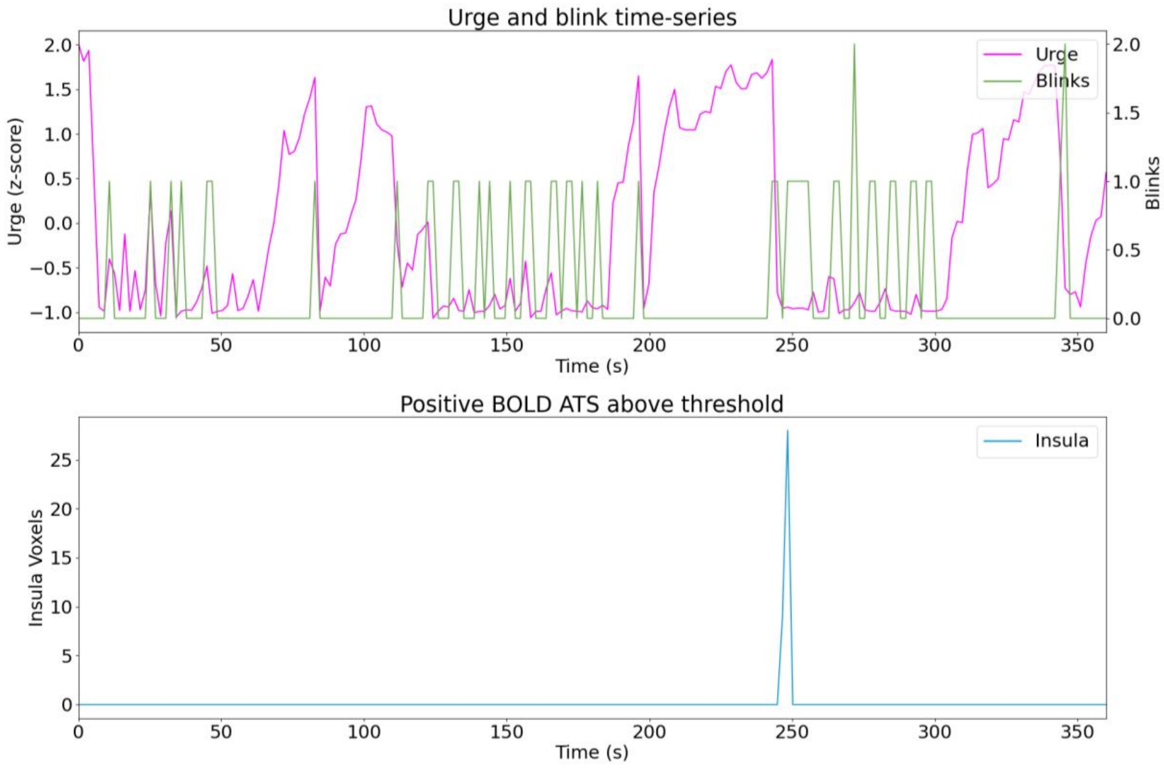

Figure SD.44. *Sub19 run01*

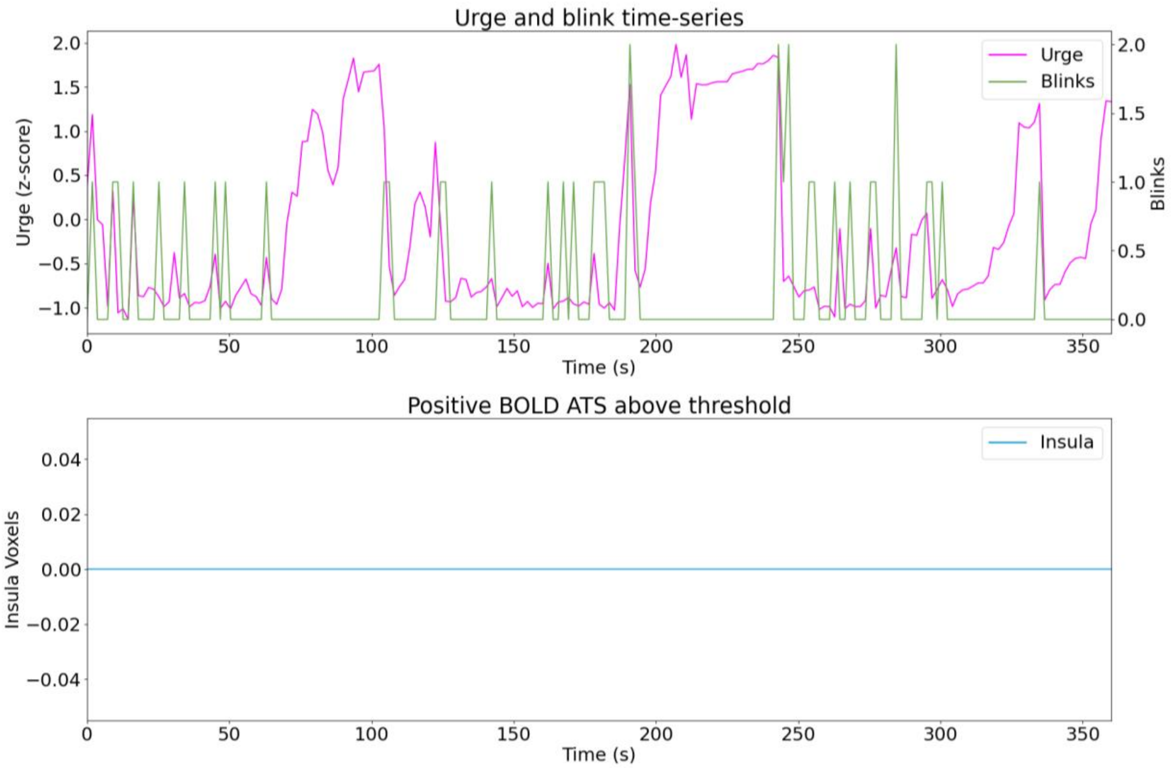

Figure SD.45. *Sub19 run03*

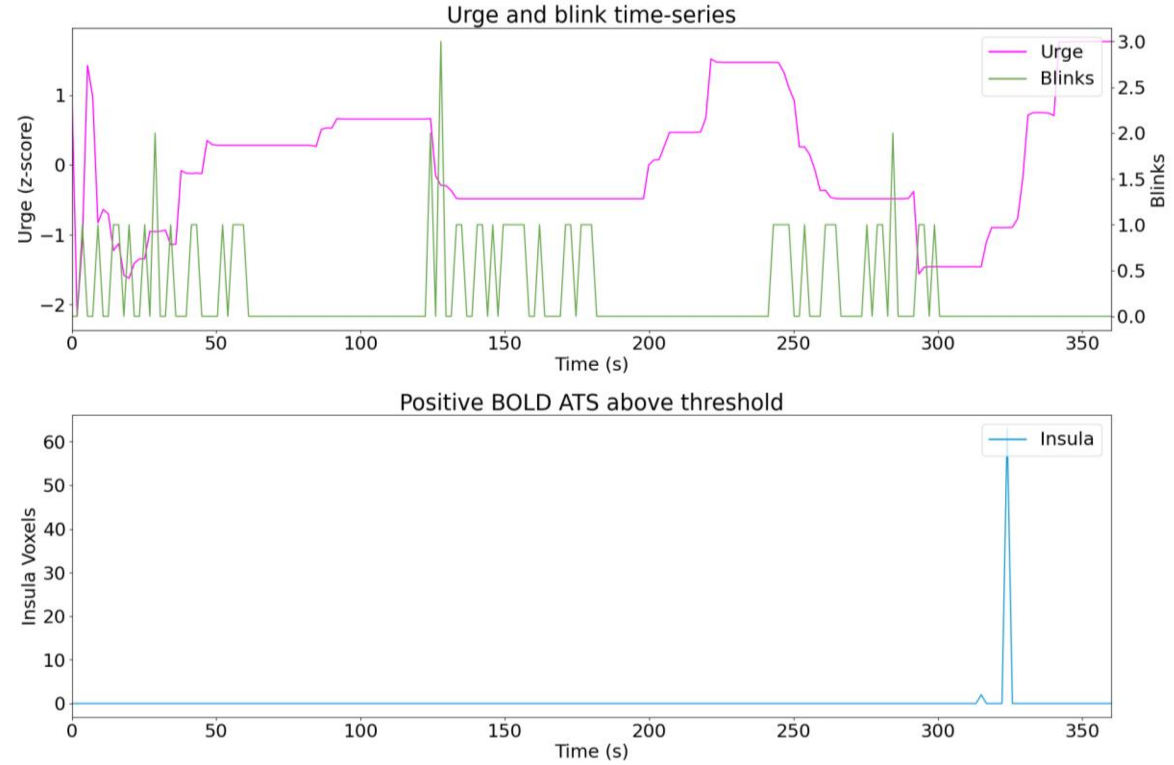

Figure SD.46. *Sub20 run01*

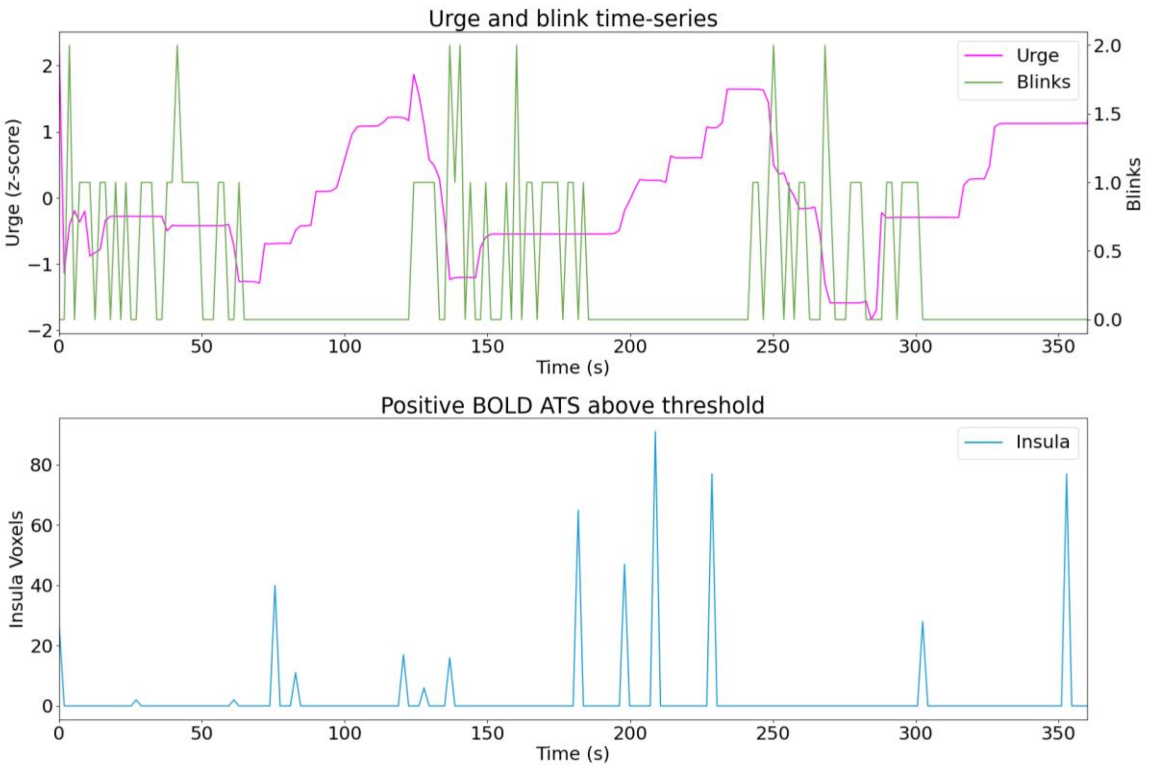

Figure SD.47. *Sub20 run02*

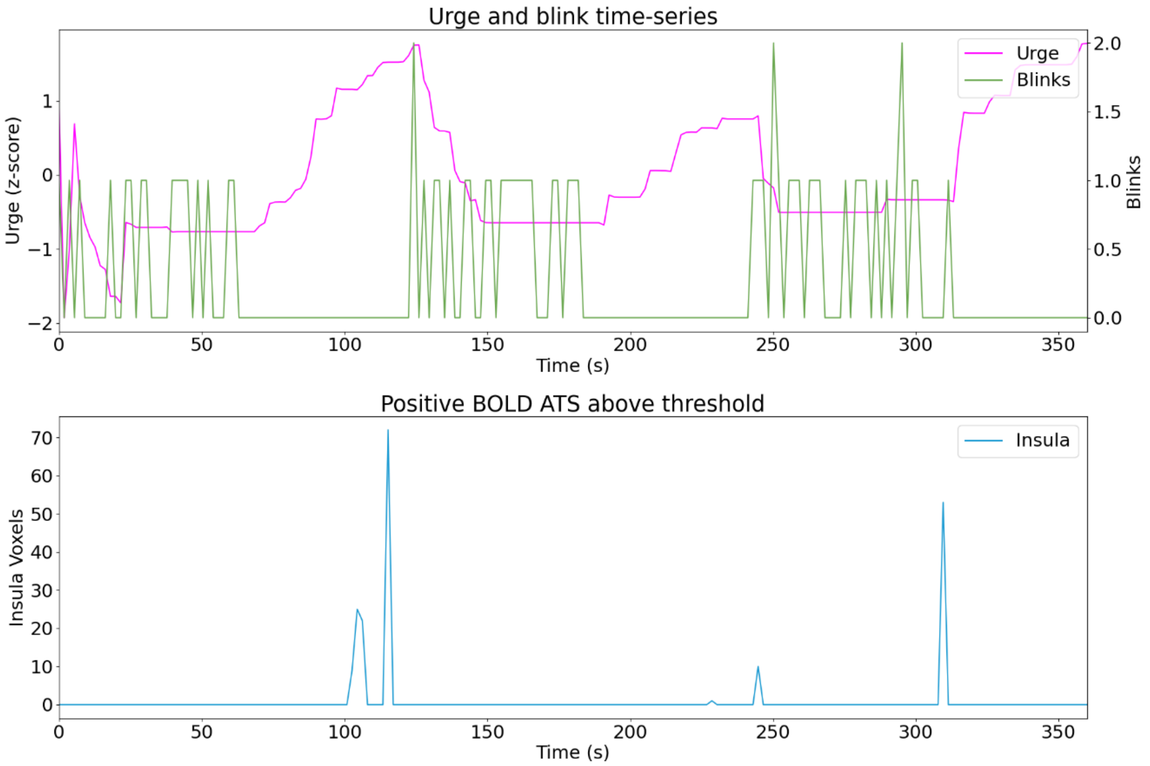

Figure SD.48. *Sub20 run03*

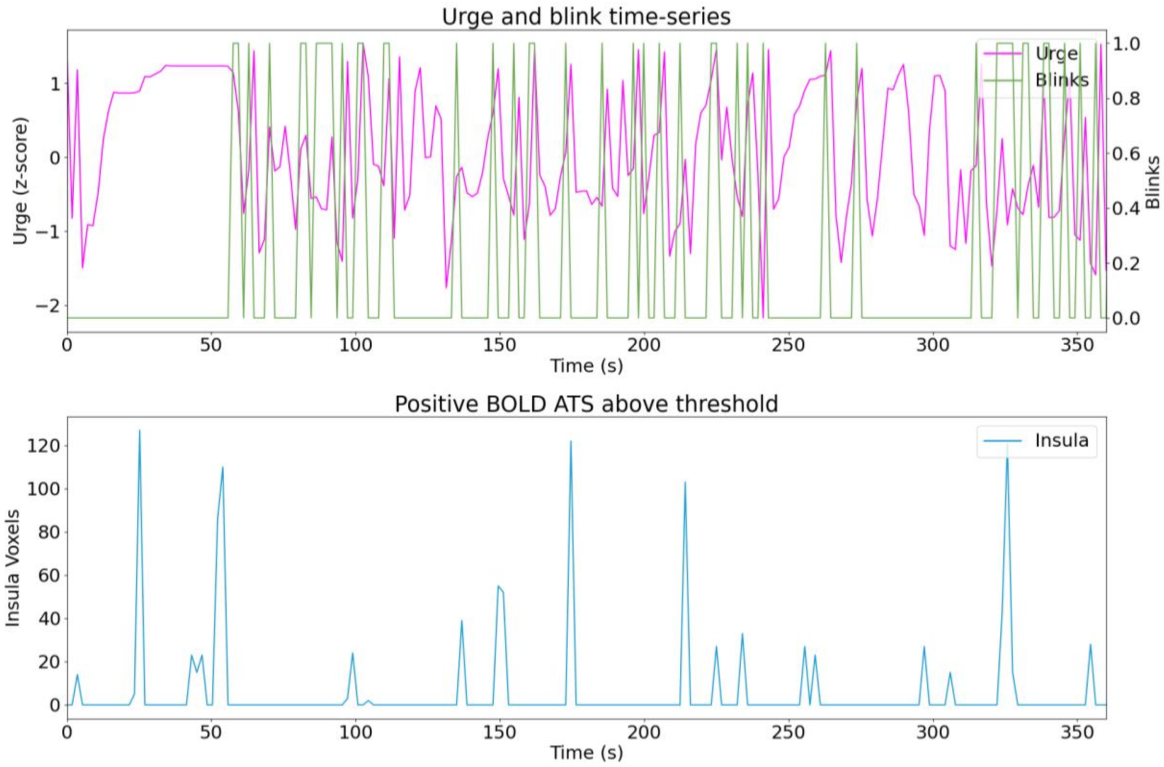

Figure SD.49. *Sub21 run03*

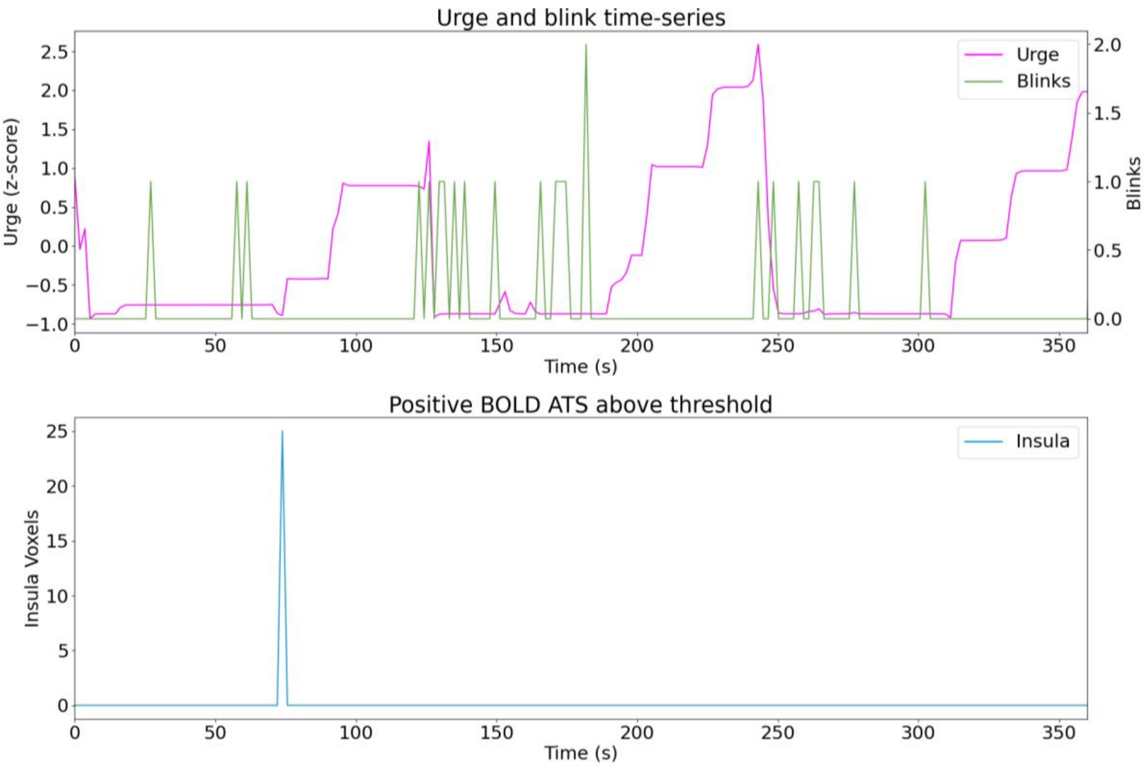

Figure SD.50. *Sub22 run01*

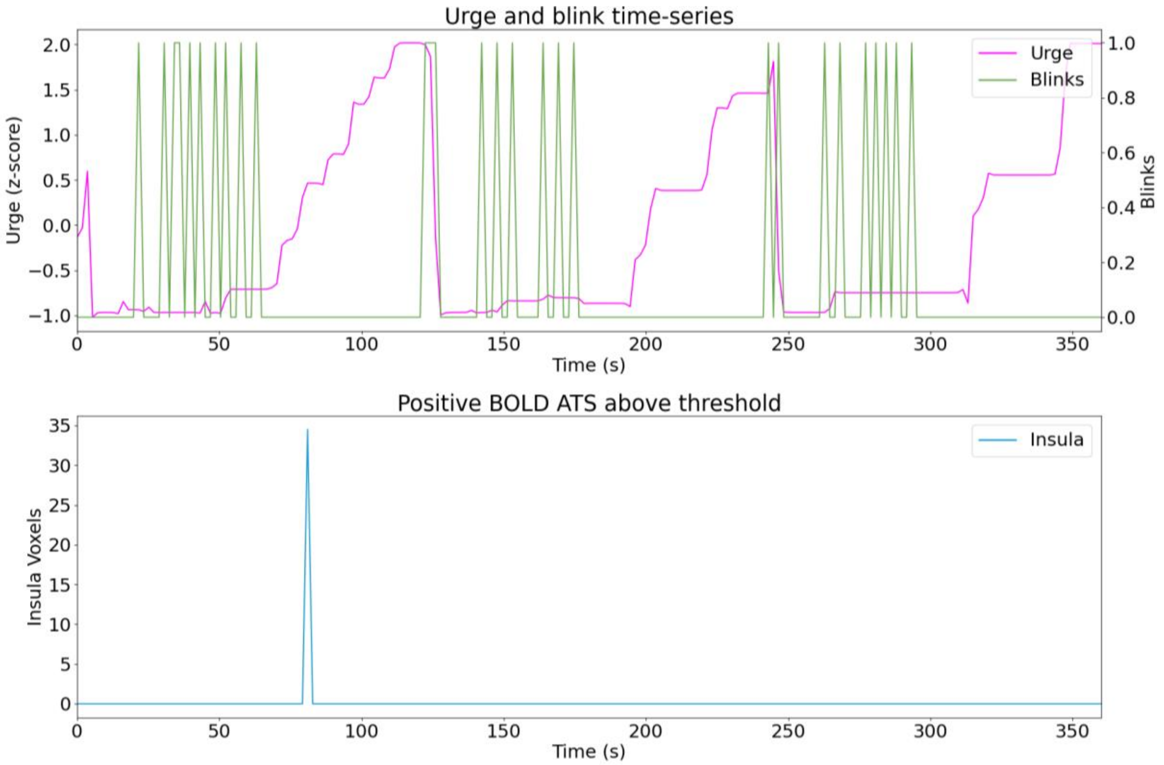

Figure SD.51. *Sub22 run02*

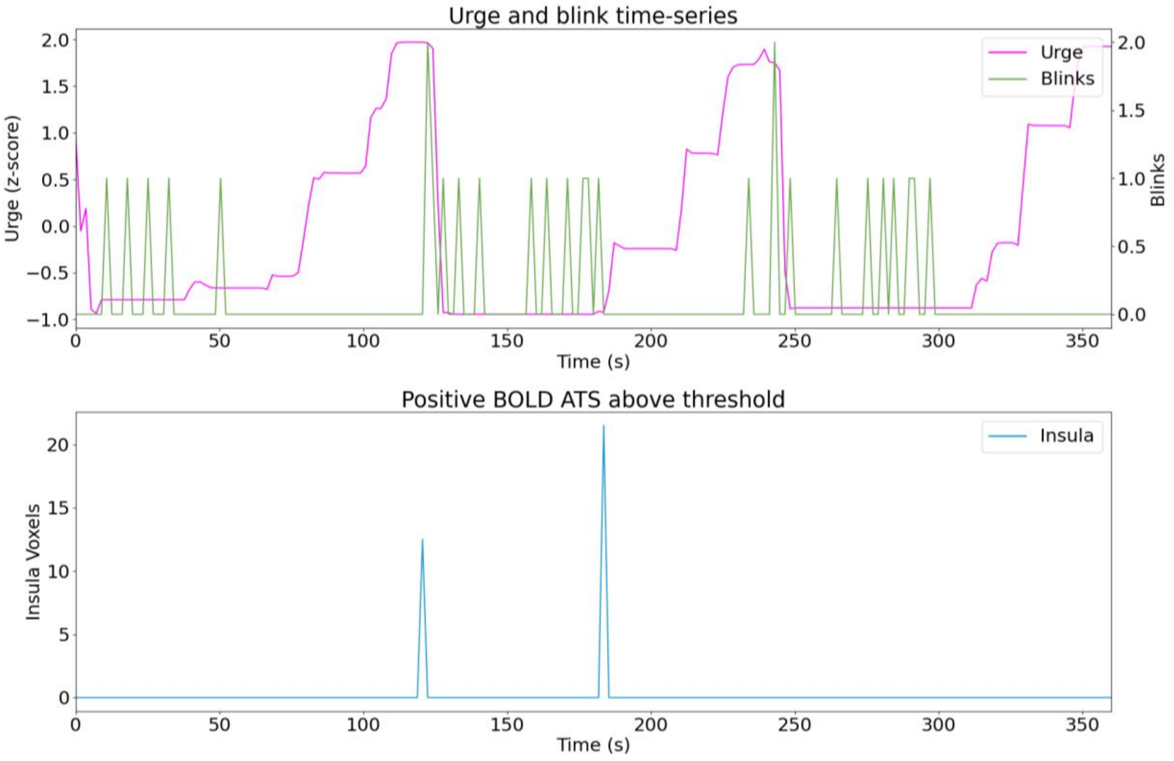

Figure SD.52. *Sub22 run03*
